# A comprehensive analysis of the genetic diversity and environmental adaptability in worldwide Merino and Merino-derived sheep breeds

**DOI:** 10.1186/s12711-023-00797-z

**Published:** 2023-04-03

**Authors:** Simone Ceccobelli, Vincenzo Landi, Gabriele Senczuk, Salvatore Mastrangelo, Maria Teresa Sardina, Slim Ben-Jemaa, Christian Persichilli, Taki Karsli, Valentin-Adrian Bâlteanu, María Agustina Raschia, Mario Andrés Poli, Gabriel Ciappesoni, Farai Catherine Muchadeyi, Edgar Farai Dzomba, Nokuthula Winfred Kunene, Gesine Lühken, Tatiana Evgenievna Deniskova, Arsen Vladimirovich Dotsev, Natalia Anatolievna Zinovieva, Attila Zsolnai, István Anton, Szilvia Kusza, Nuno Carolino, Fátima Santos-Silva, Aldona Kawęcka, Marcin Świątek, Roman Niżnikowski, Marija Špehar, Gabriel Anaya, Antonio Granero, Tiago Perloiro, Pedro Cardoso, Silverio Grande, Beatriz López de los Santos, Coralie Danchin-Burge, Marina Pasquini, Amparo Martínez Martínez, Juan Vicente Delgado Bermejo, Emiliano Lasagna, Elena Ciani, Francesca Maria Sarti, Fabio Pilla

**Affiliations:** 1 Department of Agricultural, Food and Environmental Sciences, Università Politecnica delle Marche, 60131 Ancona, Italy; 2 Department of Veterinary Medicine, University of Bari ‘‘Aldo Moro”, 70010 Valenzano, Italy; 3 Department of Agricultural, Environmental and Food Sciences, University of Molise, 86100 Campobasso, Italy; 4 Department of Agricultural, Food and Forest Sciences, University of Palermo, 90128 Palermo, Italy; 5 Laboratoire des Productions Animales et Fourragères, Institut National de la Recherche Agronomique de Tunisie, Université de Carthage, 2049 Ariana, Tunisia; 6 Department of Animal Science, Faculty of Agriculture, Eskisehir Osmangazi University, 26040 Eskisehir, Turkey; 7 Laboratory of Genomics, Biodiversity, Animal Breeding and Molecular Pathology, Institute of Life Sciences, University of Agricultural Sciences and Veterinary Medicine of Cluj-Napoca, 400372 Cluj-Napoca, Romania; 8 Instituto de Genética “Ewald A. Favret”, Instituto Nacional de Tecnología Agropecuaria, CICVyA-CNIA, B1686 Hurlingham, Buenos Aires, Argentina; 9 Instituto Nacional de Investigación Agropecuaria, 90200 Canelones, Uruguay; 10 Agricultural Research Council - Biotechnology Platform, Onderstepoort, 0110 Pretoria, South Africa; 11 Discipline of Genetics, School of Life Sciences, University of KwaZulu-Natal, 3209 Scottsville, Pietermaritzburg, South Africa; 12 Department of Agriculture, University of Zululand, 3886 Kwadlangezwa, South Africa; 13 Institute of Animal Breeding and Genetics, Justus Liebig University, 35390 Giessen, Germany; 14 L.K. Ernst Federal Research Center for Animal Husbandry, 142132 Podolsk, Russian Federation; 15 Department of Animal Breeding, Institute of Animal Science, Hungarian University of Agriculture and Life Sciences, Kaposvár Campus, 2053 Herceghalom, Hungary; 16 Centre for Agricultural Genomics and Biotechnology, Faculty of Agricultural and Food Sciences and Environmental Management, University of Debrecen, 4032 Debrecen, Hungary; 17 Instituto Nacional de Investigação Agrária e Veterinária, 2005-048 Vale de Santarém, Portugal; 18 Department of Sheep and Goat Breeding, National Research Institute of Animal Production, 32-083 Kraków, Poland; 19 Department of Animal Breeding, Institute of Animal Sciences, Warsaw University of Life Sciences-SGGW, 02-786 Warsaw, Poland; 20 Croatian Agency for Agriculture and Food, 10000 Zagreb, Croatia; 21 MERAGEM Group, Department of Genetics, University of Córdoba, 14071 Córdoba, Spain; 22 Asociación Nacional de Criadores de Ganado Merino (ACME), 28028 Madrid, Spain; 23 Associação Nacional de Criadores de Ovinos da Raça Merina (ANCORME), 7005-665 Évora, Portugal; 24 Associação de Produtores Agropecuários (OVIBEIRA), 6000-244 Castelo Branco, Portugal; 25 Associazione Nazionale della Pastorizia (ASSONAPA), 00187 Rome, Italy; 26 Departamento de Investigación y Desarrollo, EA GROUP SC, 06700 Villanueva de la Serena, Spain; 27 Institut de l’Elevage, 75595 Paris Cedex 12, France; 28 Departamento de Genética, Universidad de Córdoba, 14071 Córdoba, Spain; 29 Department of Agricultural, Food and Environmental Sciences, University of Perugia, 06121 Perugia, Italy; 30 Department of Bioscience, Biotechnology and Biopharmaceutics, University of Bari “Aldo Moro”, 70124 Bari, Italy

## Abstract

**Background:**

To enhance and extend the knowledge about the global historical and phylogenetic relationships between Merino and Merino-derived breeds, 19 populations were genotyped with the OvineSNP50 BeadChip specifically for this study, while an additional 23 populations from the publicly available genotypes were retrieved. Three complementary statistical tests, Rsb (extended haplotype homozygosity between-populations), XP-EHH (cross-population extended haplotype homozygosity), and runs of homozygosity (ROH) islands were applied to identify genomic variants with potential impact on the adaptability of Merino genetic type in two contrasting climate zones.

**Results:**

The results indicate that a large part of the Merino’s genetic relatedness and admixture patterns are explained by their genetic background and/or geographic origin, followed by local admixture. Multi-dimensional scaling, Neighbor-Net, Admixture, and TREEMIX analyses consistently provided evidence of the role of Australian, Rambouillet and German strains in the extensive gene introgression into the other Merino and Merino-derived breeds. The close relationship between Iberian Merinos and other South-western European breeds is consistent with the Iberian origin of the Merino genetic type, with traces from previous contributions of other Mediterranean stocks. Using Rsb and XP-EHH approaches, signatures of selection were detected spanning four genomic regions located on *Ovis aries* chromosomes (OAR) 1, 6 and 16, whereas two genomic regions on OAR6, that partially overlapped with the previous ones, were highlighted by ROH islands. Overall, the three approaches identified 106 candidate genes putatively under selection. Among them, genes related to immune response were identified via the gene interaction network. In addition, several candidate genes were found, such as *LEKR1*, *LCORL*, *GHR*, *RBPJ*, *BMPR1B*, *PPARGC1A*, and *PRKAA1*, related to morphological, growth and reproductive traits, adaptive thermogenesis, and hypoxia responses.

**Conclusions:**

To the best of our knowledge, this is the first comprehensive dataset that includes most of the Merino and Merino-derived sheep breeds raised in different regions of the world. The results provide an in-depth picture of the genetic makeup of the current Merino and Merino-derived breeds, highlighting the possible selection pressures associated with the combined effect of anthropic and environmental factors. The study underlines the importance of Merino genetic types as invaluable resources of possible adaptive diversity in the context of the occurring climate changes.

**Supplementary Information:**

The online version contains supplementary material available at 10.1186/s12711-023-00797-z.

## Background

Sheep (*Ovis aries*) are one of the oldest livestock species, i.e. they were domesticated by humans around 10,500 years before the present (YBP) in the Fertile Crescent (South-eastern Anatolia and the Iranian Zagros Mountains) [1, 2]. Then, sheep accompanied humans in their migrations from Asia into Europe and subsequently dispersed throughout the world [3]. One of the possible routes for the spread of the first farmers into continental Europe is the Danube valley, followed by a maritime route through the Mediterranean Sea [4–6]. Sheep provide a variety of resources, including meat, milk, and wool, and they play an essential role in the global agricultural economy since the Neolithic age [7], when they were almost exclusively reared for meat consumption and their skin. Throughout the Neolithic period and across Eurasia, there is no archaeological evidence of the use of wool, which became a known practice only later, during the Bronze Age in the Near East [8]. Later, zoo-archaeological studies have shown that sheep selected for secondary products (wool and milk) were spread throughout Europe, replacing, in many areas, the primitive sheep populations [9]. During the Bronze Age the dominant colour of the wool was brown, whereas, in the Iron Age, sheep with white fleece became widespread [10]. The oldest wool textile remains date back to 1500 YBP [11, 12].

Specialisation for wool production in the early Iron Age was well documented by the Romans [10, 11]. Roman classical literature reported that the best wool sheep originated in Southern Italy (*Tarentum*) and Greece, and were later exported to other parts of the Empire, including the Iberian Peninsula [13, 14]. The development of Spanish Merino sheep for high-quality wool production dates back to the late Middle Ages [15], when they were probably used as starting genetic material for composite stocks that may well have been influenced, across centuries, by the Phoenicians’, Romans’, and Moors’ conquests of the Iberian Peninsula [11]. In particular, a recent study by Kandoussi et al. [16], based on the analysis of the mitochondrial DNA (mtDNA) control region, provided support to the theory of a possible contribution of North African sheep to the growth of the Middle Ages Merino population. Another study [17], using genome-wide single nucleotide polymorphisms (SNPs), identified genetic patterns for intercontinental Merino populations that are compatible with a partial Italian ancestry, possibly corresponding to the documented gene flow during the Roman period. Notably, the Roman writer *Lucius Junius Moderatus Columella* [13] chronicled the complex three-way crossbreeding practice that was carried out in *Hispania Baetica* by his uncle. To improve wool quality (fine white wool), he used North African, Italian (*Tarentum*), and local sheep from the Iberian Peninsula. The resulting fine wool animals have spread over the centuries throughout the Iberian Peninsula and in other territories of the Spanish Empire. Consequently, the “Merino Breed” industry became one of the most important industries of the Iberian Peninsula and sheep were strongly protected by Royal decrees, forbidding their exportation out of the Iberian Peninsula for several centuries [18]. In the early eighteenth century, Merinos exited Spain through Kingdom agreements and smuggling. Then, Merino flocks were crossed with local breeds in many countries worldwide [19–21].

Due to centuries of selection that have led to local adaptation, sheep have thrived in a diverse range of environmental conditions along their spreading routes [22]. This is particularly evident in Merino and Merino-derived sheep, which are nowadays present in a wide variety of productive environments around the world and make them the predominant wool producing genetic type [17]. Well known for its exceptional white fleece and the abundant production of soft, fine, and curly wool, Merino sheep represent a key genetic resource thanks to their ability to face harsh climatic conditions, poor quality feeding and arid landscapes. Moreover, Merinos display an extraordinary ability to adapt and perform in an extensive production system, in a wide range of environmental conditions spanning from the Mediterranean to the Continental European and Asian climates as well as subtropical, and both warm and cold arid lands [23–25]. To date, the genetic determinism of Merino adaptability has been poorly investigated while relevant efforts have been made to identify the loci involved in wool and production traits [26–29]. However, since the Merino and Merino-derived breeds are genetically related but widespread in very different environments, they provide an interesting model to investigate the genetic differences related to environmental adaptation.

Moreover, Merino and Merino-derived breeds played a crucial role in the economic development of several countries, such as Australia, New Zealand, and Uruguay, through the marketing of high-quality wool. Conversely, the European Merino and Merino-derived sheep suffered a significant numerical decrease, with many of these breeds now considered to have reached an endangered status [17]. Over the past decades, several genetic population studies have been performed to explore the patterns of variability and population structure of the Merino and Merino-derived breeds. Different approaches have been adopted to analyse their genetic diversity, especially based on molecular markers, such as microsatellites and mtDNA [30–32], SNP arrays [33–36] and whole-genome resequencing data [29, 37]. Recent papers have contributed to the reconstruction of the Merino history and the so-called worldwide phenomenon of “Merinization”, defining the genetic basis and underlying the specific Merino phenotype, by using genome-wide SNP data [17, 38, 39]. However, these studies do not include several of the local populations. Because of this gap, the relationships between these local Merino and Merino-derived sheep and other breeds existing worldwide have not been addressed and clarified, and a comprehensive description of the distribution of the diversity of present-day Merino and Merino-derived sheep breeds is still lacking. The aim of the present investigation was to enhance the knowledge about the historical and phylogenetic relationships by studying a representative collection of Merino and Merino-derived sheep breeds at a global level. A fine-scale analysis of a broad array of Merino and Merino-derived breeds is deemed necessary to confirm the evolutionary pathway, the overall genetic diversity, the breed structure, the breeding strategies or safeguard programmes, and the uniqueness of the Merino gene pool. In addition, three approaches for the detection of signatures of selection: extended haplotype homozygosity between-populations (Rsb), cross-population extended haplotype homozygosity (XP-EHH), and runs of homozygosity (ROH) were applied to identify the mechanisms that drive the genetic differences between Merino types adapted to different environmental features.

## Methods

### Dataset construction and sample collection

Blood samples were collected from animals that were as unrelated as possible based on farmers’ knowledge, and flock-book, when possible. Genomic DNA was extracted using a GenElute Blood Genomic DNA kit (Sigma Aldrich, St. Louis, MO, USA). DNA quality and quantity were determined using a NanoDrop 2000c spectrophotometer (Thermo Scientific, Wilmington, DE, USA). High-quality samples (i.e., having DNA concentrations of at least 50 ng/µL and A260/280 ratios of ~ 1.8) were then subjected to SNP array genotyping.

In total, 1694 individuals belonging to 42 Merino and Merino-derived sheep breeds were selected. All the samples were genotyped using the OvineSNP50 BeadChip (Illumina, San Diego, CA). Chromosomal coordinates for each SNP were obtained from the OAR v4.0 reference genome assembly [40]. Twenty-one breeds were genotyped specifically for this study (including 3 breeds partially resampled and re-genotyped), whereas the genotyping data of the remaining 21 breeds were extracted from previous studies [17, 19, 20, 33–35, 41].

The number of samples and the breed origin of the genotyped animals are provided in Additional file 1: Table S1. The Spanish Merino breed consists of approximately 140,000 animals registered in the flock-book (SME_FB) that is managed by the competent authority (Asociación Nacional de Criadores de Ganado Merino—ACME). However, the Spanish Merino breed, due to its maternal genetic characteristics, has been used for crossbreeding with other Merino and Merino-derived breeds to improve meat production, and currently in Spain, there are approximately 2.4 million sheep with a morphological pattern close to the Spanish Merino. The Spanish Merino-type population (SME_TP) is a heterogeneous population ranging from 100% Merino animals from a genetic point of view to animals with signs of different levels of crossbreeding with other breeds.

### Data quality control

The PLINK software v1.9 was used for data management and quality control [42]. The newly generated samples were combined with published genotypes (see Additional file 1: Table S1), applying the PLINK *--merge* command. The datasets were combined using only SNPs with unique ID and chromosomal positions as identified by the SNPchiMp v.3 software [40].

The combined dataset was filtered to retain loci or individuals that meet the following criteria: (1) SNPs with a call rate (CR) higher than 0.90, (2) SNPs with a chromosomal or physical location, (3) animals with a frequency of missing genotypes lower than 0.05, and/or (4) SNPs with a minor allele frequency (MAF) higher than 0.01, (5) SNPs that were on autosomes, and (6) animals for which the pair-wise identity-by-state (IBS) between genotypes (based on all markers) was less than 0.99. In addition, six duplicated samples were detected and excluded from further analyses. After quality control, 28,662 SNPs and 1644 animals remained for further analysis.

### Genetic diversity

Prior to the analysis of the patterns of genetic variation and population structure, to mitigate the effects of linkage disequilibrium (LD) between loci the dataset was pruned using the PLINK function *--indep-pairwise 50 5 0.5*, resulting in 27,552 SNPs. For each breed, observed (H_O_) and expected (H_E_) heterozygosities were estimated using PLINK, and the historical trends in effective population size (*N*_*e*_) were estimated using the SNeP software [43] and setting default values and a correction to adjust for LD and small sample sizes. The most recent and distant estimates of *N*_*e*_ were taken 13 generations back (*N*_*e13*_), and 50 generations back (*N*_*e50*_), respectively. Prieur et al. [44] have reported that the 50K SNP BeadChip is not suitable for estimating the *N*_*e*_ more than 100 generations back. Since the large differences in population size within the combined dataset could negatively affect the estimations by the SNeP software, the number of analysed individuals per breed was fixed at a minimum of 20. Breeds with fewer animals were discarded (see Additional file 2: Table S2), while breeds with a sample size larger than 20 were sub-sampled using the approach implemented in the BITE R package [45].

### Genetic relationship and population structure

Multi-dimensional scaling (MDS) plots based on pairwise IBS distances were generated using the BITE R package [45] at both the single individual and population levels to investigate the relationships within and between breeds.

To assess reticulate relationships between populations, the Reynolds’ distances were calculated using the Arlequin v. 3.5.2.2 software [46] and subsequently visualised via a Neighbor-Net graph with the SplitsTree v. 4.14.5 software [47]. The same software was used for the computation of pairwise *F*_ST_ values and their respective statistical significances (*P* < 0.05) with 10,000 permutations. The output was visualised using an in-house R script. Population structure was assessed by the model-based approach implemented in the Admixture software v1.3 [48] and plotted using the *membercoef.circos* function in the R package BITE [45]. Analyses were run for *K* values ranging from 2 to 45 (corresponding to the number of breeds, plus three additional *K* values to account for possible sub-structuring within breeds). To identify the best fitting number of hypothetical populations, for each *K* value, both cross-validation error values and the number of iterations needed to reach convergence were considered.

Finally, to explore the genetic relationship and migration events among the breeds, a maximum likelihood dendrogram was generated using the TREEMIX software [49]. For this analysis, the genotyping data of Sardinian White sheep (SAW) [17] were used as an outgroup, and migration edges from 1 to 10 were allowed. The most predictive number of migration edges was selected using the *optM* function in the R package OptM [50]. The results were then plotted using the *plot_tree* function in the R package BITE [45].

### Runs of homozygosity

Analysis of high-homozygosity regions across the genome, i.e. runs of homozygosity (ROH), was conducted for each animal using PLINK. The dataset, without LD pruning, consisting of 28,662 SNPs, was used to estimate ROH according to the criteria described by Mastrangelo et al. [51]: the minimum length was set to 1 Mb (*--homozyg-kb*), and one missing SNP and up to one possible heterozygous genotype were allowed in the ROH (*--homozyg-window-missing 1* and *--homozyg-window-het 1*), the minimum number of consecutive SNPs included in a ROH was set to 30 (*--homozyg-snp 30*), the minimum SNP density per ROH was set to one SNP every 100 kb (*--homozyg-density 100*), and the maximum gap considered between consecutive homozygous SNPs was 500 kb (*--homozyg-gap 500*).

The inbreeding coefficient based on ROH (*F*_ROH_) for each animal was calculated as follows:

$$F_{{{\text{ROH}}}} = L_{{{\text{ROH}}}} /L_{{{\text{AUT}}}}$$

where *L*_ROH_ is the total length of the ROH in the genome of an individual and *L*_AUT_ is the specified length of the autosomal genome covered by the SNPs on the chip (2452.06 Mb). For each breed, the mean number of ROH (MN_ROH_) and the average ROH length (AL_ROH_) were estimated. In addition, each ROH was categorised based on its physical length into five classes: 1 to < 5 Mb, 5 to < 10 Mb, 10 to < 15 Mb, 15 to < 20 Mb, and ≥ 20 Mb, as previously reported by Purfield et al. [52].

### Detection of signatures of selection

To examine the effects of contrasting environments on the genomic architecture of Merino and Merino-derived sheep breeds, two sheep groups reared in different climate zones (as defined by the Köppen–Geiger classification system) were selected. The first group included breeds that are reared under a temperate or Mediterranean climate: Gentile di Puglia (GDP), Sopravissana (SOP), Trimeticcio di Segezia (TRS), Merino Branco (MBA), Merino de Beira Baixa (MBB), Spanish Merino (SME_FB), and Spanish Merino type (SME_TP). The second group included breeds that are reared under cold or Continental climate: Hungarian Merino (HUG), Merinolandschaf (MLA), Polish Old Type Merino (POM), Soviet Merino (SOV), Stavropol (STA), Transylvanian Merino (TRM). Two extended haplotype homozygosity (EHH)-derived statistics [53] based on LD were used to detect long homozygous stretches of the genome with high frequencies of particular haplotypes generated by selective sweeps between the two Merino groups: (i) the standardized log-ratio of the integrated site-specific EHH between pairs of populations test (Rsb) [54]; and (ii) the cross-population EHH test (XP-EHH) [53].

The analyses were computed using the *rehh* package in R [55]. Haplotypes were reconstructed from the genotyped SNPs using fastPHASE [56]. Since fastPHASE is based on haplotype clusters, with a size that should be set a priori, the toolkit implemented in the *imputeqc* R package [57] was used to estimate the optimal number of haplotype clusters (K) needed for haplotype phasing. The *Imputeqc* package is designed to assess the imputation quality and/or to choose the model parameters for imputation. In the current study, K = 30 provided the best imputation quality (for 5% of masked data). Therefore, this value was used to run fastPHASE. Considering that Rsb and XP-EHH values are normally distributed, a Z-test was applied to identify significant SNPs under selection. Two-sided *P*-values were derived as p*Rsb* = − log10[1–2|Φ(*Rsb*) − 0.5|] and p*XP-EHH* = − log10[1–2|Φ(*XP-EHH*) − 0.5|], where Φ (x) is the Gaussian cumulative distribution function. To detect signatures of selection, the genome was split into 250-kb sliding windows that partially overlapped 10 kb with adjacent windows. A window is classified as putatively under selection when it contains at least three SNPs exceeding the significance threshold of − log10 (*P*-value) = 4.

Genomic regions that were characterised by a high frequency of ROH occurrence (ROH islands) were identified. To do this, the number of times each SNP occurred in a ROH was considered and normalised by dividing it by the number of animals included in the analysis. The top 0.999 SNPs of the percentile distribution of the locus homozygosity range within each group were considered as potential ROH islands, as suggested in previous studies [58, 59].

Genomic regions identified by the three approaches for detecting signatures of selection were interrogated for genes annotated to the OAR v4.0 genome assembly using the Genome Data Viewer provided by NCBI [60]. To investigate the biological function and the phenotypes that are known to be affected by each annotated gene, a comprehensive search in the available literature and public databases, including information from other species, was carried out. Furthermore, a gene network analysis was performed by adopting GeneMANIA [61], using the *Homo sapiens* datasets. This tool enables the construction of weighted interaction networks, which use as a source a very large set of functional association data including protein and genetic interactions, pathways, co-expression, co-localisation, and protein domain similarity. For a more detailed description of the considered network categories see [62].

## Results

### Dataset

During quality control of the initial raw dataset, 44 individuals with low-quality genotypes and six duplicated individuals were excluded. Thus, the working version of the dataset included 1644 animals and 42 populations with an average population size of 40.26 and a size ranging from 10 (Macarthur Merino—MCM, South Africa Mutton Merino—SMM) to 105 (Corriedale Uruguay—COU). All the animals included in the analysis had high-quality genotyping. Additional file 1: Table S1 provides a summary of the pre- and post-quality control attributes of the working dataset.

### Genetic diversity

Descriptive statistics of the genetic diversity are in Table 1. The observed heterozygosity (Ho) across breeds, ranged from 0.243 ± 0.209 (Merino de Rambouillet—RAM) to 0.426 ± 0.108 (Merino Argentina—MAR) with an overall mean of 0.375 ± 0.150. A similar trend was observed for the expected heterozygosity. As expected, RAM and MCM had the lowest genetic diversity, due to genetic drift, which is consistent with their significantly smaller population size and the fact that the RAM flock is closed since the nineteenth century.

**Table 1:** Genetic diversity indices for the Merino and Merino-derived breeds

Breed	Code	H_O_ ± SD	H_E_ ± SD	*N*_*e13*_	*N*_*e50*_	*F*_*ROH*_
Australian Industry Merino	AIM	0.387 ± 0.119	0.393 ± 0.111	103	292	0.030
Arapawa	APA	0.341 ± 0.147	0.362 ± 0.137	63	174	0.134
Australian Poll Merino	APM	0.393 ± 0.117	0.394 ± 0.110	97	284	0.024
Australian Merino	AUM	0.383 ± 0.125	0.392 ± 0.113	104	300	0.038
Berrichon du Cher	BDC	0.322 ± 0.195	0.321 ± 0.189	–	–	0.099
Chinese Merino	CME	0.387 ± 0.158	0.372 ± 0.129	83	231	0.033
Corriedale Argentina	COA	0.392 ± 0.137	0.385 ± 0.120	91	262	0.026
Corriedale Uruguay	COU	0.377 ± 0.134	0.376 ± 0.127	72	197	0.046
Dohne Merino	DHM	0.381 ± 0.143	0.375 ± 0.128	89	232	0.032
Merinofleischschaf	FLE	0.373 ± 0.176	0.358 ± 0.141	–	–	0.032
Gentile di Puglia	GDP	0.388 ± 0.118	0.393 ± 0.111	98	303	0.031
Groznensk	GRZ	0.404 ± 0.128	0.398 ± 0.105	114	350	0.006
Hungarian Merino	HUG	0.386 ± 0.127	0.386 ± 0.116	102	286	0.023
Ile de France	IDF	0.357 ± 0.170	0.361 ± 0.166	89	194	0.053
Kyrgyz Mountain Merino	KMM	0.400 ± 0.141	0.386 ± 0.117	83	252	0.010
Merino Argentina	MAR	0.426 ± 0.108	0.532 ± 0.292	–	–	0.020
Merino Branco	MBA	0.377 ± 0.138	0.385 ± 0.118	105	297	0.046
Merino de Beira Baixa	MBB	0.379 ± 0.128	0.390 ± 0.107	109	346	0.058
Macarthur Merino	MCM	0.244 ± 0.234	0.230 ± 0.200	–	–	0.295
Merinizzata Italiana	MEI	0.388 ± 0.153	0.402 ± 0.160	95	278	0.031
Merino d’Arles	MER	0.394 ± 0.158	0.391 ± 0.143	–	–	0.016
Merinolandschaf	MLA	0.382 ± 0.156	0.372 ± 0.130	82	224	0.038
Merino Uruguay	MUR	0.393 ± 0.133	0.382 ± 0.122	86	248	0.022
Paska	PAK	0.387 ± 0.147	0.384 ± 0.120	97	307	0.026
Palas Merino	PAL	0.393 ± 0.157	0.378 ± 0.126	88	248	0.024
Polish Colored Merino	PCM	0.353 ± 0.179	0.338 ± 0.156	65	154	0.072
Polish Merino	POL	0.378 ± 0.158	0.371 ± 0.131	92	247	0.034
Polish Old Type Merino	POM	0.372 ± 0.163	0.36 ± 0.1390	83	213	0.032
Merino Preto	PRE	0.390 ± 0.142	0.391 ± 0.114	102	315	0.032
Merino de Rambouillet	RAM	0.243 ± 0.209	0.237 ± 0.200	48	77	0.237
American Rambouillet	RMB	0.365 ± 0.126	0.376 ± 0.124	103	270	0.051
Salsk	SAL	0.395 ± 0.135	0.388 ± 0.114	101	295	0.016
South Africa Merino	SAM	0.371 ± 0.142	0.378 ± 0.126	95	252	0.055
Spanish Merino type	SME_TP	0.383 ± 0.117	0.396 ± 0.107	107	331	0.039
Spanish Merino (flock-book)	SME_FB	0.387 ± 0.140	0.392 ± 0.112	106	329	0.032
South Africa Mutton Merino	SMM	0.334 ± 0.221	0.307 ± 0.174	–	–	0.097
Sopravissana	SOP	0.386 ± 0.141	0.408 ± 0.155	92	289	0.030
Soviet Merino	SOV	0.401 ± 0.164	0.389 ± 0.123	–	–	0.007
Stavropol	STA	0.395 ± 0.162	0.386 ± 0.127	–	–	0.020
Turkish Merino	TKM	0.399 ± 0.140	0.392 ± 0.112	105	332	0.010
Transylvanian Merino	TRM	0.386 ± 0.157	0.378 ± 0.125	81	244	0.036
Trimeticcio di Segezia	TRS	0.374 ± 0.164	0.368 ± 0.134	–	–	0.065
Mean		0.375 ± 0.150	0.380 ± 0.136			

H_O_ observed heterozygosity, H_E_ expected heterozygosity, *N*_*e1*3_ and *N*_*e50*_ effective population size estimated for 13 and 50 generations back (only for breeds with a number of analysed individuals ≥ 20), *F*_*ROH*_ genomic inbreeding coefficients in each breed

Values of the recent effective population size (*N*_*e13*_) ranged from 114 (Groznensk—GRZ) to 48 (RAM) and a similar trend was found for the *N*_*e50*_ values, with the highest value for GRZ (350) and the lowest for RAM (77). However, SNeP analysis identified a marked reduction in *N*_*e*_ from 50 to 13 generations back for the GDP, GRZ, Paska (PAK), SOP, MBB, Merino Preto (PRE), SME_FB, SME_TP, and Turkish Merino (TKM) breeds. The decline in *N*_*e*_ for RAM, Arapawa (APA), and Polish Colored Merino (PCM) was less steep compared to that for the other breeds, probably due to their small population sizes (Table 1).

In total, 24,201 ROH with lengths ranging from 2.54 to 881.34 Mb were identified based on 1644 individuals. Of these, 1539 individuals had at least one ROH, resulting in an average of 16.55 ROH per individual and a number of ROH ranging from 2.59 (GRZ) to 89.80 (MCM) (see Additional file 3: Table S3). The average length of ROH across breeds was 7.22 and ranged from 5.59 Mb for the Merinofleischschaf (FLE) to 9.33 Mb for the APA breed. Analysis of the distribution of ROH according to size highlighted that, for all the populations/breeds, most of the detected ROH were shorter than 10 Mb, with a few long ROH exceeding 20 Mb. FLE, GRZ and Ile de France (IDF) had a larger portion of their genome covered in short ROH (1 to 10 Mb), whereas APA and TRS showed ROH longer than 20 Mb (see Additional file 3: Table S3).

Individual genomic inbreeding was evaluated using ROH analysis, as reported in Table 1 and Additional file 4: Fig. S1. The highest mean value of *F*_ROH_ is observed in MCM (0.295), followed by RAM (0.237) and APA (0.134), while the lowest values are observed in the Russian breeds (GRZ, 0.006; SOV, 0.007; Kyrgyz Mountain Merino—KMM, 0.010) and in TKM (0.010).

### Breed divergence and structure

**Fig. 1:**
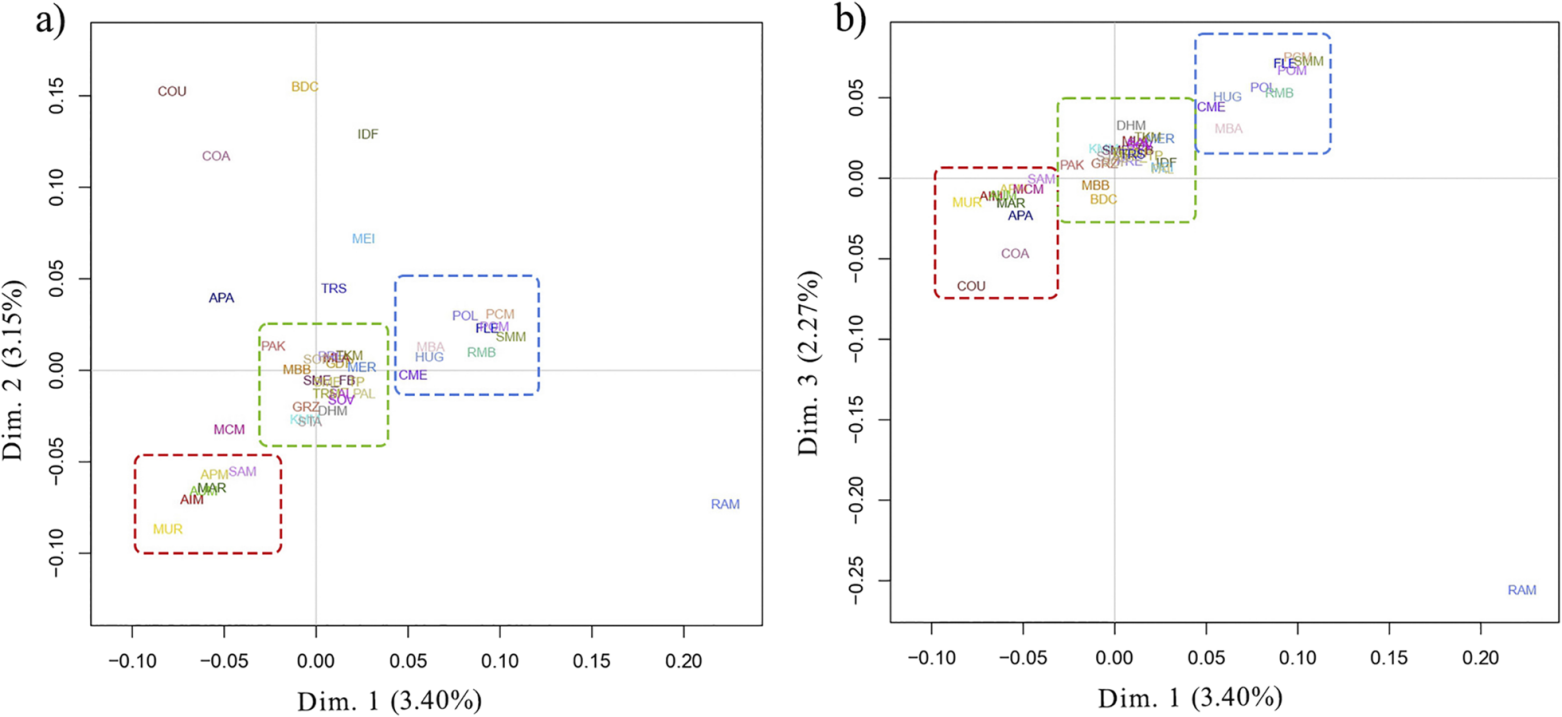
Multi-dimensional scaling plots of the Merino and Merino-derived sheep breeds. Dimension 1 vs 2 (**a**) and dimension 1 vs 3 (**b**). The different colours of the squares reflect the three clusters that include breeds with similar genetic background. For a full definition of breeds see Table 1.

To examine the genetic relationships between breeds, MDS plots of the pairwise IBS distances (Fig. 1) were generated by comparing the first vs the second dimensions (Fig. 1a) and the first vs the third dimensions (Fig. 1b).

The first dimension of the MDS plot accounted for 3.40% of the genetic diversity, the second dimension for 3.15%, and the third dimension for 2.27%. In this analysis, three principal clusters according to breed genetic background or geographical proximity are highlighted. All the Australian breeds (Australian Industry Merino—AIM, Australian Poll Merino—APM, and Australian Merino—AUM) clustered together and near the South American breeds (MAR and Merino Uruguay—MUR) and the South Africa Merino sheep (SAM). Chinese Merino (CME), American Rambouillet (RMB), Polish Merinos (Polish Merino—POL, POM, and PCM), HUG, SMM, and FLE clustered close to each other. All the other European Merino and Merino-derived sheep clustered together, except for Merinizzata Italiana (MEI) and TRS. These last two breeds are known to have been influenced by the IDF and MLA breeds, and indirectly by the Berrichon du Cher (BDC) breed, as confirmed by their position in the MDS plot close to these French breeds. Other breeds (APA, Corriedale Argentina—COA, COU, BDC, IDF, and RAM) were scattered over the plot. The MDS plot representing single animals (see Additional file 5: Fig. S2) emphasised the central position of Spanish and European breeds, and suggests genetic introgression of Australian, French, and German strains in most of the Merino-derived breeds.

The relationships between the studied sheep breeds were assessed by calculating a pairwise *F*_ST_ matrix and Reynolds’ genetic distances (see Additional file 6: Table S4 and Additional file 7: Fig. S3), which corroborate some of the MDS results. *F*_ST_ for all pairs of breeds differed significantly from 0 (*P* < 0.05) and ranged from 0.004 to 0.411, with the closest pair-wise value (0.004) observed between the Australian AIM and AUM breeds. RAM and MCM sheep are the most differentiated breeds, probably due to strong genetic drift effects.

**Fig. 2:**
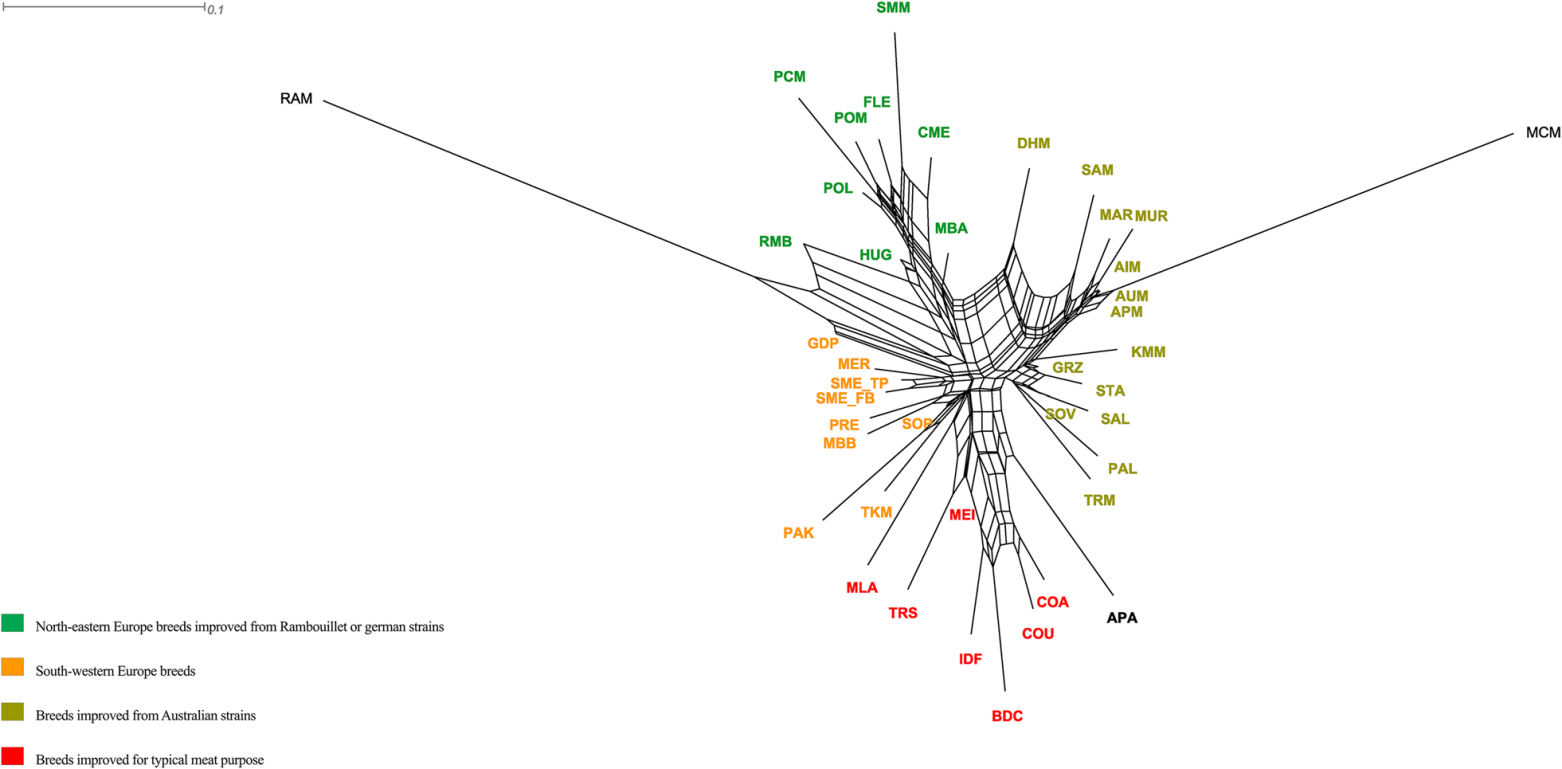
Neighbor-Net graph based on between-breed Reynolds’ distances for the whole dataset. For a full definition of breeds see Table 1

To further investigate the genetic relationships among the studied Merino sheep breeds, a Neighbor-Net graph was constructed using the Reynolds’ distances (Fig. 2).

In Fig. 2 four distinct groups can be recognised based on genetic origin and/or their geographical proximity. Accordingly, South-western European breeds were grouped in one distinct cluster, while breeds from North-eastern Europe clustered in another. The Neighbor-Net graph highlighted two additional clusters, namely (i) breeds influenced by Australian Merino strains and (ii) breeds with a typical meat or double purpose, which were found scattered in the MDS plot. The RAM, APA and MCM breeds were distinct and had long branch lengths.

**Fig. 3:**
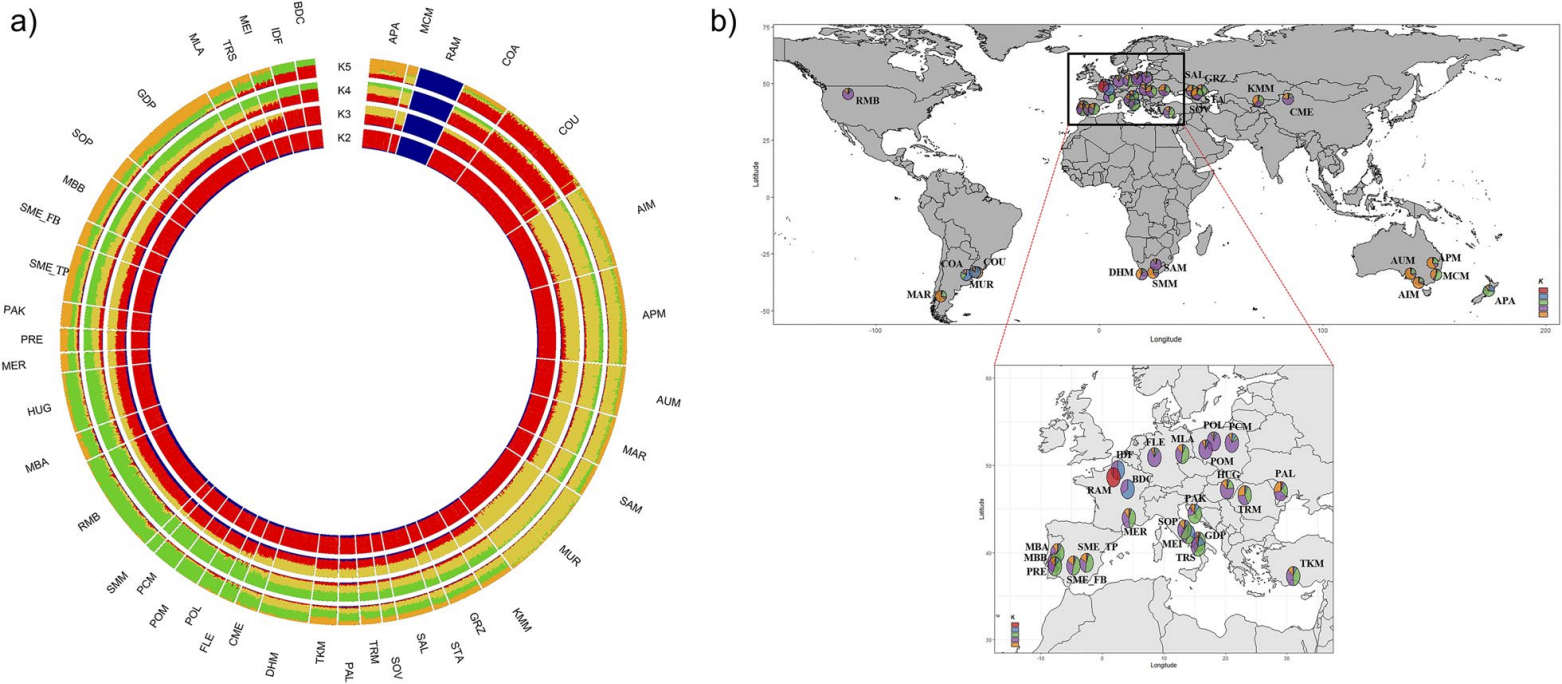
Admixture software results in a circular fashion for *K* (number of clusters) = 2 to 5 (**a**), and geographic distribution of the genomic components corresponding to the *K* = 5 resolution (**b**). For a full definition of breeds see Table 1

To determine the proportion of shared ancestry genomic components, Admixture analysis on the whole dataset was done to separate breeds according to their genetic background and/or geographic origin (Fig. 3).

The cross-validation error registered the lowest value at *K* = 43, suggesting that this value is the most probable number of clusters explaining the variation in this dataset (see Additional file 8: Fig. S4). However, admixture patterns, obtained at *K* ranging from 2 to 5 were first analysed, since they contributed to a better understanding of the ancestry of the investigated breeds. The graph presented in Fig. 3a shows the early separation of RAM (in blue, at *K* = 2). The two Corriedale (COA and COU) are separated at *K* = 3, and their cluster (in red) is mainly shared with BDC and IDF, while it is poorly shared with the three Australian breeds, the Argentinean, the Uruguayan and the South African Merino and Merino-derived breeds. These breeds clearly appear, from *K* = 3 to *K* = 5, as a rather homogeneous group, well differentiated from the others. At *K* = 4, a new cluster (in green) appears, which represents mainly some European Merino-derived breeds (FLE, HUG, POL, POM, PCM, and MBA), the Turkish Merino (TKM), the Chinese Merino (CME), the two South African breeds (SMM and Dohne Merino—DHM) and the North American RMB.

The genetic pattern at *K* = 5 was highly consistent with the Neighbor-Net graph. As expected, the genomic architecture reflected both the geographic origin and the genetic background of each breed, with some unique genetic signatures, such as those observed for the South-western European breeds, the South African breeds, and the South American improved breeds. Other breeds, such as the APA, displayed a clear sub-structuring. At *K* = 43, several breeds showed a complex admixture-like pattern with a mosaic of different genetic components (see Additional file 9: Fig. S5). Evidence of breed admixture was identified in several breeds such as: HUG, CME and POL, which are mainly admixed with FLE; the SME_FB and SME_TP share a genetic component with the Portuguese Merinos (MBB, MBA, and PRE), SOP and Merino d’Arles (MER); and the Romanian breeds (Palas Merino—PAL and TRM), which share a common genetic ancestry with Russian breeds (SOV, STA, GRZ, and Salsk—SAL). The fine wool Australian breeds (AIM, APM, and AUM) and the South American Merino breeds (MAR, MUR, COA, and COU) show a complex ancestry. It is also important to point out that the higher *K* values revealed similar genetic patterns in the two Spanish samples. In contrast, some breeds seemed to be homogeneous and clearly separated from all the others, i.e., COA, SAM, POM, PCM, SMM, RAM, MCM, MLA, IDF, and BDC.

**Fig. 4:**
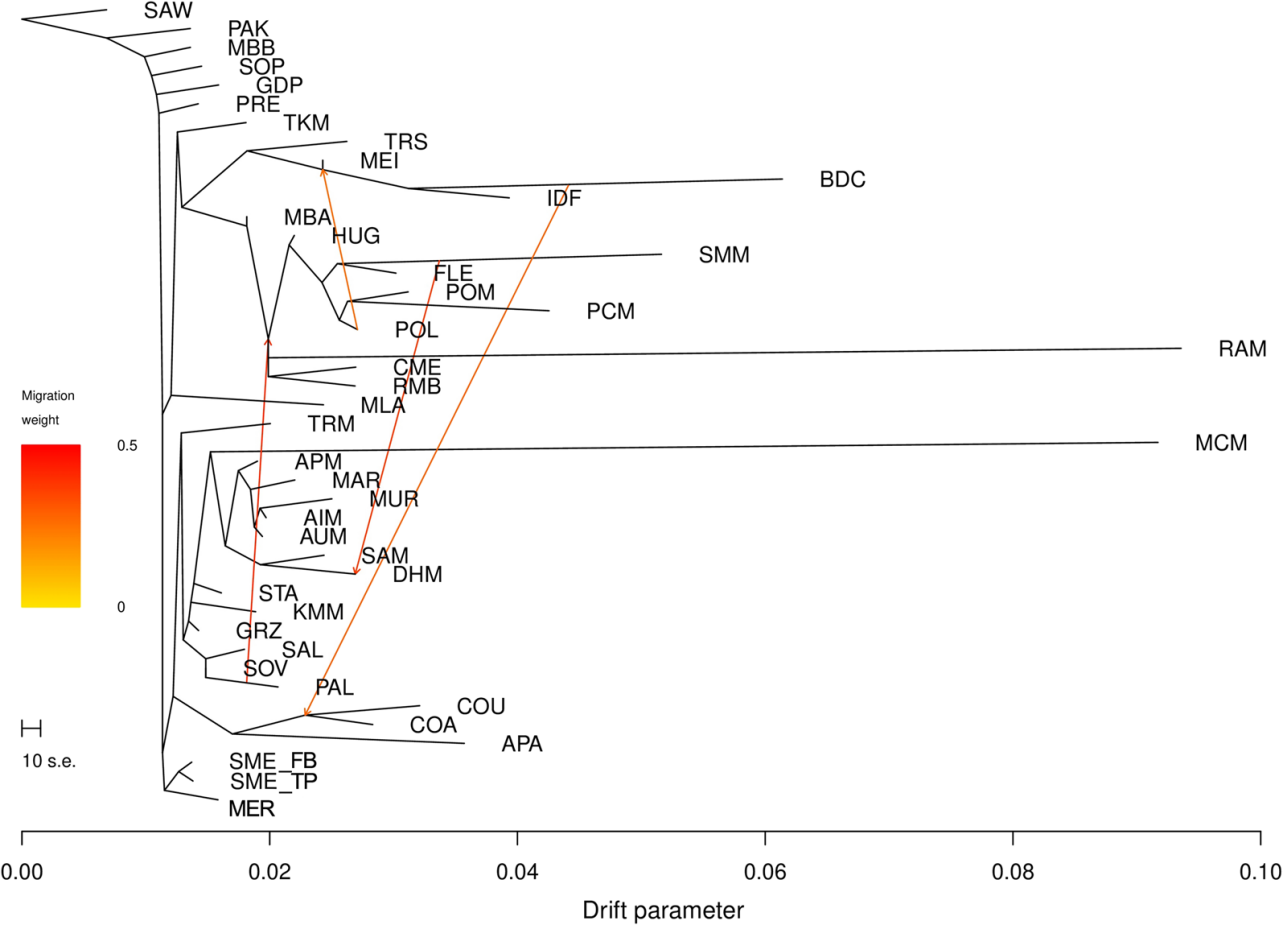
Maximum likelihood tree constructed with TreeMix when four migration events were allowed. Migration arrows are coloured according to their weight. For a full definition of breeds see Table 1

The maximum likelihood phylogenetic tree inferred by using TreeMix confirmed several of the findings already revealed by the MDS and the Admixture analyses (Fig. 4).

The linear method implemented in the *optM* function indicated a major changing point in the log likelihood at four migration events (see Additional file 10: Fig. S6). Two stronger mixture events were inferred, one between SMM and DHM, and the other connecting the North-eastern European Merino breeds with SOV. Two additional migration edges, although with a weaker signal of admixture, are highlighted between BDC and the node including the Corriedale group, as well as a migration from POL to MEI.

### Signatures of selection

**Fig. 5:**
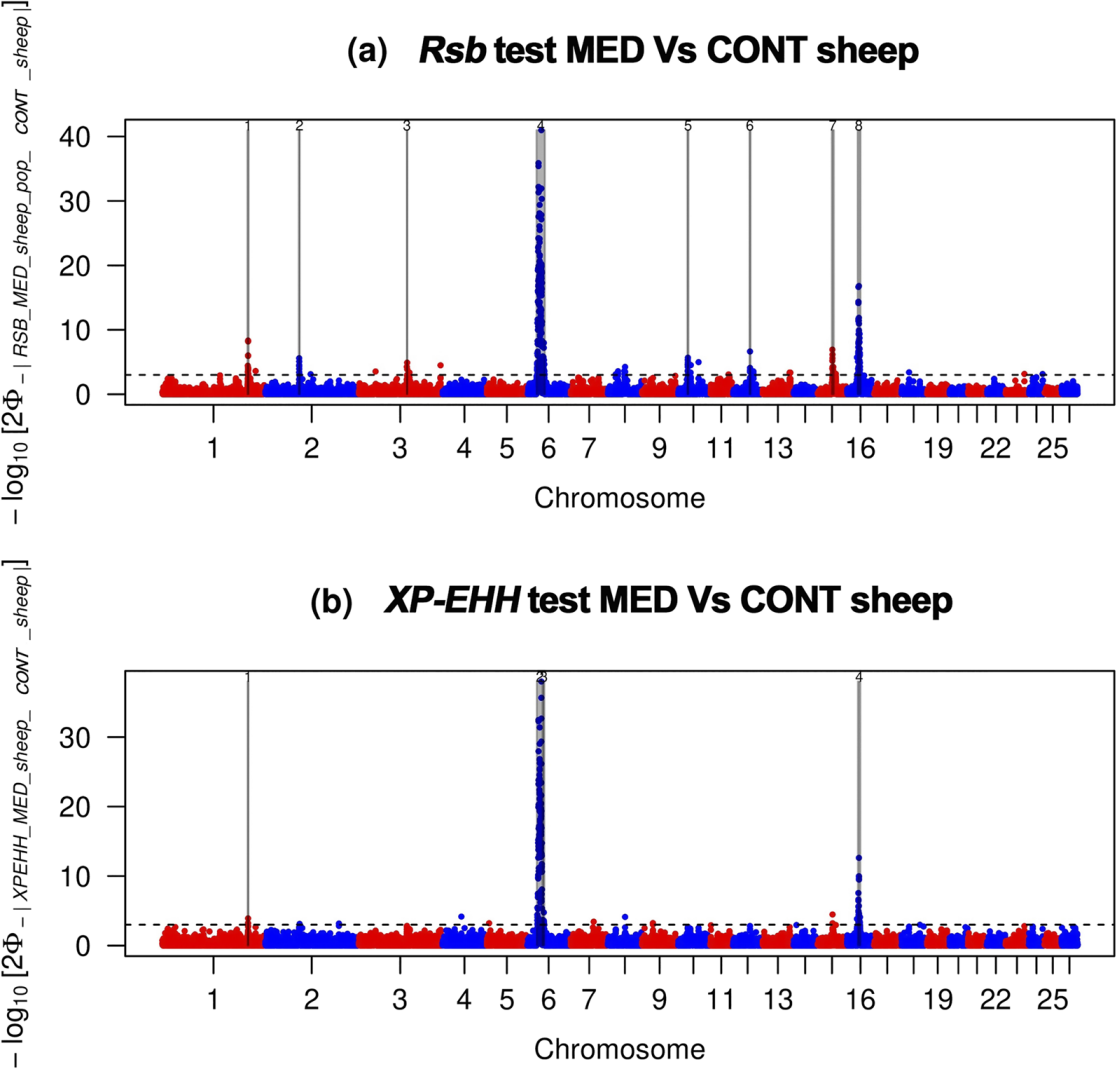
Manhattan plots showing the results of Rsb and XP-EHH tests for the autosomes in the Merino and Merino-derived breeds. **a** Rsb test for Mediterranean vs Continental Merino and Merino-derived breeds. **b** XP-EHH for Mediterranean vs Continental Merino and Merino-derived breeds. Horizontal dashed lines mark the significance threshold applied to detect the outlier SNPs (−log10 (*P* value) = 4)

The Rsb approach detected 597 SNPs that were putatively under selection (Fig. 5a). These SNPs defined eight candidate regions. The signal on OAR6 identified with Rsb (between 25.7 and 48.05 Mb) revealed 169 SNPs above the significance threshold. The XP-EHH test yielded fewer outlier SNPs than the analysis based on the Rsb approach, with 316 SNPs putatively under selection (Fig. 5b), which defined four candidate regions. As for Rsb, the candidate region on OAR6 showed the strongest signal, with 116 SNPs exceeding the significance threshold.

Four genomic regions located on OAR1, 6, and 16, ranging in size from 2.75 Mb (on OAR1) to 15.15 Mb (on OAR6), were identified by both the Rsb and XP-EHH approaches (see Additional file 11: Table S5). The strong signals corresponding to the two overlapping outlier windows on OAR6 (between 26.50 and 41.65 Mb and between 43.75 and 46.70 Mb) suggest that these regions represent potentially decisive evidence of selection processes. The four identified candidate regions harboured 106 known genes and 60 uncharacterised genes (see Additional file 11: Table S5). The main biological functions of the known genes are summarised in Additional file 12.

Six genomic regions that frequently appeared in a ROH were identified in the two groups. Additional file 13: Table S6 provides the chromosome, the start and end positions of ROH islands. ROH islands were distributed on OAR5, 6, 10, and 12 in the Mediterranean group, whereas they were only on OAR6 for the Continental group. There were overlaps between genomic regions identified with the ROH approach and those detected with the two extended haplotype homozygosity (EHH)-derived statistics. Two regions on OAR6 between 32.38 and 34.56 Mb for the Continental group and between 38.96 and 40.15 Mb for the Mediterranean group, were identified jointly by all statistical approaches. These regions harboured eight genes (*SLIT2*, *LOC101122950*, *PACRGL*, *KCNIP4*; *CCSER1*—alias *FAM190A*, *TRNAW-CCA*, *LOC105615447*, and *LOC106991208*, respectively), and their main biological functions are presented in Additional file 14.

The results of the gene network analysis for the genes located in the putatively selected regions mentioned above are presented in Additional file 15: Fig. S7 and Additional file 16: Table S7.

## Discussion

It is assumed that the first documented sheep (*Ovis aries*) bred in the world, the Merino, developed in the Iberian Peninsula, mainly in Spain [18, 63]. Several authors have addressed the question of when and how the Merino breed developed, and suggested that the origin of the populations involved in the construction of the first Merino animals is complex [11, 13, 64]. From the eighteenth century onwards, the Merinos spread all over the world and currently represent the most abundant sheep breed and the principal source of the world’s wool supply [17]. Although a previous study has already explored the genetic variability of a limited number of breeds belonging to the Merino trunk [17], the present investigation provides a broader overview of the genomic architecture in a larger sample with a wide geographic distribution.

### Genetic diversity

The results indicate that almost all the Merino-derived sheep breeds share a common evolutionary history. The study shows clear homogeneity among registered (SME_FB) and non-registered (SME_TP) animals, a high similarity to the Portuguese Merino Branco, Merino de Beira Baixa and Preto, and a central position (in the MDS plot) supporting its role as a base of the European South-western breeds. The two Spanish sample batches clustered together in the Neighbor-Net graph (Fig. 2), showing one of the shortest *F*_ST_ distances (0.006) (see Additional file 6: Table S4). Moreover, these two samples displayed a similar fragmentation in the Admixture analysis (see Additional file 9: Fig. S5). The results of our study are not consistent with the recognisable differences within historical flocks found by Granero et al. [39]. In that study, the Spanish Merinos were investigated at the country level, with the animals belonging to the Flock Book of the Merino breed (SME_FB) considered separately, while the commercial flocks (SME_TP) showed admixture with other Merino breeds. However, our results should be treated with care, since the Merino-type population is very large and heterogeneous in Spain.

A large part of the Merino’s historical patterns of admixture and their genetic relatedness worldwide is explained by their genetic background and/or geography, followed by local admixture (Fig. 3b). Indeed, in many cases, they represent a mixture of indigenous breeds crossed with other Merino strains as a strategy to achieve higher yields in wool and meat and to give robust performance under conditions of challenging production environments, diseases, extreme climate, and poor nutrition [65].

The heterozygosity measured in the Merino and Merino-derived breeds was relatively high (mean 0.38) and similar to the values reported in southern and western European sheep breeds [17, 19]. A low diversity, high level of inbreeding, and large mean number of ROH segments were found for MCM and RAM (Table 1), which are characterised by a small population size, geographic isolation and founder effects [17]. In fact, MCM was developed in the early nineteenth century mainly from English Merino, in a closed nucleus flock. The RAM breed has a unique history: it was introduced to Rambouillet (France) from Spain in the late eighteenth century, and then was maintained without any introgression; only rams were sold to French farms and other countries around the world. Since the nineteenth century, pedigree and recording performance of RAM have been rigorously controlled to minimise its inbreeding rate [66]. Interestingly, both ancient and recent inbreeding events have had an impact on the genome of the APA breed, as highlighted by the length distribution of ROH. APA is the only breed that is feral and unmanaged [67], and it probably originated from multiple introduction events and admixture of genetically differing sources in New Zealand [17]. Also, in the Neighbor-Net graph, long branches for APA, MCM, and RAM were highlighted and might reflect extensive genetic drift and inbreeding.

The results of the MDS plot, Neighbor-Net, and Admixture analyses are consistent with known breeds histories and broad geographic classifications. The MDS analysis shows a clear gradient along the first component (Fig. 1 and see Additional file 5: Fig. S2), that separate wool-type specialised breeds from dual purpose breeds. This genetic pattern was already observed by Ciani et al. [17].

Neighbor-Net and Admixture graphs suggest a common ancestry between Australian fine-wool breeds, South American (MAR, and MUR), Eastern Europe (Russian and Romanian) and South African sheep (Figs. 2 and 3). As in other countries of South America, Spanish sheep were introduced into Argentina and Uruguay in the sixteenth century. In the early nineteenth century, these populations called “criollos” were improved with Saxon, Negrete and Rambouillet Merinos, with the aim to produce better carcasses and finer wool, and only later were the local populations crossed with rams from Australia [68]. Today, rams or semen from Australia are frequently introduced into South America, and for this reason, the Uruguayan Merino sheep are usually referred to as Australian Merino.

In Eastern Europe, most of the Russian fine-wool breeds were developed during the Soviet period by crossing local breeds with Australian Merino and American Rambouillet rams [34, 69]. The Romanian PAL and Transylvanian TRM Merino breeds were developed at the beginning of the nineteenth century, when local sheep were crossed with Merino de Rambouillet and Merino Precoce in the first instance, and Russian and Australian Merinos later on [70]. After 1990, the purpose of sheep rearing in Romania was changed to meat and/or milk production [71]. An event of migration was detected between PAL and RAM (Fig. 4). This result is consistent with several previous studies that suggested a contribution of specialised French sheep breeds that were used via crossbreeding schemes to increase production-related traits, carcass, and meat quality of lambs [72, 73].

The history of the South African Merino stretches back to 1789, when the Dutch Government donated two Spanish Merino rams and four Spanish Merino ewes to Col. Jacob Gordon, the military commander in the Cape at that time. The introduction of other Merino strains, most notably those from Australia, occurred during the last 200 years [74]. Maximum likelihood assessment based on TreeMix analysis (Fig. 4) provided a detailed insight into the population history of the DHM breed. It was created through intensive selection and interbreeding of South African Merino ewes and German Mutton Merino rams (commonly known as South African Mutton Merino) [75], with the objective of improving the breed’s robustness and maintaining good production performance for meat and wool traits [35]. The small amount of genetic divergence (see Additional file 6: Table S4) and a strong mixture event revealed by the TreeMix analysis confirmed a common ancestry between DHM and SMM.

In the Neighbor-Net graph (Fig. 2), SMM clustered with a group that includes North-eastern European breeds that were improved with Rambouillet and or/German strains. These results support findings reported in the literature on the origin of the SMM breed from German Merino imported in the 1930s into South Africa and then selected as a dual-purpose wool and meat sheep breed [76].

In Turkey, the TKM breed shows a pattern of admixture that was quite similar to that observed for DHM, which supports the hypothesis that they share a German common ancestor (Fig. 3). In fact, it is well-known that TKM originated by crossbreeding between German Mutton Merino and White Karaman sheep, which is the commonly reared native sheep breed in Turkey [77].

Both Neighbor-Net (Fig. 2) and Admixture graphs (see Additional file 9: Fig. S5) highlighted that the genetic material of POL and HUG shared some similarity, which could result from genetic introgression from a common ancestor. Peter et al. [78] observed in their study a short genetic distance between the two aforementioned breeds. HUG and POL were developed by crossing RAM, FLE, Russian Merinos, Merino Precoce, and Australian Merinos with the local population [36, 79, 80]. The TreeMix analysis also hinted at a possible gene flow from POL to European Mutton Merino breeds (MEI, IDF, and BDC) (Fig. 4). This can be explained by the fact that BDC rams are very often used in multi-breed crossing schemes with Polish Merino sheep with the aim of improving the slaughter qualities of F1 lambs [81]. Interestingly, the Admixture analysis identified a unique genetic pattern in the PCM breed (see Additional file 9: Fig. S5), which is consistent with its origin dating back to the 1980s in the Experimental Station of the National Research Institute of Animal Production in Kołuda Wielka (Poland). The aim was to produce a variety of colourful Polish Merino. The creation of this breed by selecting only coloured individuals out of Polish Merino herds, probably resulted in a founder effect [36].

In the MDS plot and the Neighbor-Net graph (Figs. 1 and 2), the RMB breed grouped with the North-eastern European breeds. After importation of Rambouillet rams from France in the second half of the eighteenth century, the RMB breed became a dual-purpose breed in the U.S. [82]. The American Rambouillet contributed to the development of many other Merino-derived breeds, such as the Chinese Merino [17]; this fact was also confirmed by genetic overlaps detected in the MDS plot and Admixture analysis (Figs. 1 and 3).

A clear genetic closeness of all the South-western European Merino breeds was revealed by the low *F*_ST_ values (see Additional file 6: Table S4), the joint clustering in the Neighbor-Net graph (Fig. 2) and similar Admixture patterns (Fig. 3). Interestingly, MER appeared near to SME_FB and SME_TP (Fig. 2), with a similar genetic pattern (see Additional file 9: Fig. S5). This result can be explained by different scenarios that describe how this breed may have originated. According to history, the Merino d’Arles resulted from a cross between a local sheep breed and Spanish Merino of the “Imperial and Royal Bergerie d’Arles” established by the Napoleonic administration in the 1800s. In addition, it is known that, in several flocks of Merino d’Arles, Spanish, Portuguese, and French Merino Précoce have been used to improve its stature. Another possible scenario could be that the Merino d'Arles derived from Spanish Merino animals reared in Roussillon before that province was annexed to France under the terms of the “Peace of the Pyrenees”, also called “Treaty of The Pyrenees”, (Nov. 7, 1659), that ended the Franco-Spanish War of 1648–59 [83].

Both Admixture patterns (Fig. 3 and see Additional file 9: Fig. S5) and the Neighbor-Net graph (Fig. 2) show a common genetic background for the Merino in Portugal and the Spanish Merinos, thus it can be assumed that the sheep in Portugal came from Spain because of the geographic proximity between the two countries and of the transhumance routes of Spanish Merino herds. Moreover, both Spain and Portugal constituted the same kingdom between 1580 and 1640, and this coincides with the expansion of the Merino breed in the Iberian Peninsula [84]. After the nineteenth century, new breeds, which originated from the Spanish Merinos (Merino Precoce, RAM, and IDF), were imported to improve the herds in Portugal [85]. Nevertheless, breeds such as the Portuguese MBB, and PRE and the Italian SOP, and GDP, which are assumed to have been crossbred with Merino, still have traces of their ancestral genetic backgrounds, as suggested by the Admixture (see Additional file 9: Fig. S5) and TreeMix analyses (Fig. 4). Interestingly, the Italian SOP and GDP breeds could also be part of the ancestral Merino gene pool, which has been well documented by Roman writers [13, 14].

The Mutton Merino breeds (COA, COU, BDC, IDF, MEI, TRS, and MLA) highlight a pattern of admixture that is quite similar (Fig. 3 and see Additional file 9: Fig. S5). They are also grouped in a defined cluster (Fig. 2), probably due to their common origin from the British gene pool. The Corriedale sheep represents a composite breed that resulted from crossbreeding between Merino and the British Lincoln and was originally developed in New Zealand. This breed has been exported to Australia and many countries, and makes up the largest population of all sheep in South America. In Uruguay in 1970, the Corriedale population was estimated at about 8.5 million, or about half the national herd [86]. In Argentina, Corriedale is a dual-purpose breed raised in the North-eastern region of the country, valued for both wool and meat production [41]. BDC and IDF share a common history. In the nineteenth century, some breeders cross-bred BDC with British breeds, such as the Cotswold, Dishley Leicester, Romney and Southdown, with the aim of improving its meat quality. In addition, since in the 1830s the IDF breed was developed by crossing Dishley Leicester and Rambouillet, it shares some genetic background with British breeds. Other breeds, such as TRS and MEI have an overlapping genetic history since they derive from local population crosses with European Mutton Merino imported into Italy at various times [32, 87].

### Detection of signatures of selection

The three methods used to detect signatures of selection highlighted two jointly supported candidate regions on OAR6 (32.38–34.56 Mb and 38.96–40.15 Mb). These regions include four genes (*CCSER1*, *SLIT2*, *PACRGL*, and *KCNIP4*), which are known or assumed to be involved in inflammatory and immune responses (*SLIT2* and *KCNIP4*) and in growth traits (*CCSER1*, *PACRGL*, and *KCNIP4*) (see Additional file 14).

Overall, the three approaches identified more than 100 genes that were putatively under selection in the considered sheep groups. The resulting gene interaction network pointed to gene functions that are related to immune response (“*Regulation of humoral immune response*”, “*Humoral immune response*”). The most supported gene function (“*Pore complex*”) has been also clearly demonstrated to play a role in antiviral innate immunity [88]. Similarly, “*Complement activation*” is known to represent a crucial mechanism in the innate defence against common pathogens [89]. Another supported gene function was “*Integral component of plasma membrane*”. Integral membrane proteins have been assigned a broad range of functionalities, which include roles as transporters, linkers, channels, receptors, enzymes, structural membrane-anchoring domains, proteins involved in accumulation and transduction of energy, and proteins responsible for cell adhesion. Also among these proteins are toll receptors, which are known to play an essential role in the recognition of microbial components [90] and class I cytokine receptors, which are responsible for cell proliferation and fate decisions of immune and hematopoietic cells [91]. “*Positive regulation of protein serine/threonine kinase activity*” was also found among the detected gene functions. Serine/threonine kinases play a role in the regulation of cell proliferation, apoptosis, cell differentiation, and embryonic development. Moreover, nuclear Dbf2-related (NDR) serine/threonine kinases have been shown to regulate cytokine-induced inflammation [92]. “*Negative regulation of leukocyte migration*” was another supported gene function inferred from the gene interaction network. Leukocyte migration is a fundamental immune response that is a prerequisite to the entry of effector cells such as neutrophils, monocytes, and effector T cells to sites of infection [93].

Based on the evidence that gene functions related to immune response were identified by the gene interaction network, it is tempting to speculate that the two groups of investigated Merino and Merino-derived sheep breeds (reared under Mediterranean vs Continental climate) have mainly experienced differential environmental-driven selection pressure. This would also be consistent with the fact that, overall, candidate genes related to metabolism, immunity, hypoxia, and temperature were found in this study (see Additional files 12 and 14). Different climates may influence differentiation mainly due to temperature, but they could also indirectly affect the incidence and impact of infection agents and metabolic status. In this regard, it has been reported that native Mediterranean sheep breeds (Altamurana and Gentile di Puglia) of the Apulian region are resistant to tick-borne diseases (TBD) compared to sheep from Northern Europe [94]. This aspect is probably also true for sheep breeds that experience environments with climatic and pedological characteristics similar to those of Apulia. This hypothesis needs to be validated by further studies and data on appropriate phenotypes.

Moreover, it is well-known that a close relationship exists between hypoxia and physiological or pathological immune activity [95]. In addition, we found several candidate genes relevant for growth, body size and conformation traits, for which a connection with the identified candidate genes affecting metabolic status seems to be predictable. A differential anthropic impact on growth, body size and conformation traits cannot be excluded and may have occurred in the two considered breed groups, mainly through past crossbreeding practices that were aimed at improving traits connected with meat production of Merino-derived breeds. As such, the identified signatures of selection may reflect genetic contributions by local sheep breeds with which the Merinos were crossed. The results of this study also indicate a potential strong influence of a difference in availability of feed biomass between seasons in the two targeted geographical macro-areas. The identified signs of adaptative introgression may also reflect genetic contributions by local sheep breeds, which are adapted to specific environments and productive systems, with which the Merinos were crossed. Although adaptive introgression generally refers to the movement of alleles from one species to another, the introgression of adaptive alleles can also occur through crossbreeding [96]. This fact could have a great adaptive significance in the Merino-derived breeds with regard to local environmental conditions. In addition, it has been demonstrated that gene flow can promote local adaptation but also that adaptive polymorphisms can be conserved within populations in spite of a high gene flow [97].

## Conclusions

The findings from this study provide an in-depth picture of the genetic relationships between the Merino and Merino-derived breeds from a global perspective. Past and present genetic management schemes have favoured gene flow between Merino and Merino-derived breeds. This has created and maintained a high level of total genetic diversity, with the known exception of Merino de Rambouillet and Macarthur Merino, which represent small breeds characterised by a loss of genetic diversity due to genetic drift and inbreeding effects. The current study confirms, as already observed in previous analyses, the complex pattern of the genetic variability in the Spanish Merino populations, combined with the existence of specific genetic Merino strains with different aptitudes. Thus, this study highlights the role of the whole Merino population in Spain as an important genetic reservoir for future breeding programmes. In addition, the analyses provide clues about possible selection pressures, which are mainly associated with the effects of environmental factors, on the immune response. Taken together, the above-mentioned concepts highlight the potential of Merino and Merino-derived breeds that are reared in widely different environmental conditions, as useful reservoirs of possible adaptive diversity that could be linked to the current context of global environmental changes.

## Supplementary Information

**Additional file 1: Table S1.** Name of the breeds, breed codes, continent and geographic origin, sampling coordinates, environmental and main purpose, number of novel samples, number of individuals pre-quality control, post-quality control, and source of genotyping data.

**Additional file 2: Table S2.** List of populations excluded from the SNeP software analysis due to a small sample size (number of individuals < 20).

**Additional file 3: Table S3.** Descriptive statistics and classification by length of the ROH for each Merino and Merino-derived sheep breed.

**Additional file 4: Figure S1.** Distribution of ROH inbreeding coefficients for each Merino and Merino derived breed.

**Additional file 5: Figure S2.** MDS plots of Dimensions 1 vs 2 (panel a) and 1 vs 3 (panel b). Each point represents a single individual. The correspondence between breeds and symbols is given in the legend box in the upper right corner. For full definition of breeds see Table 1.

**Additional file 6: Table S4.** Matrix of pairwise estimates of *F*_ST_ statistic (above the diagonal) and pairwise Reynolds' distances between breeds (below the diagonal).

**Additional file 7: Figure S3.** Heatmap of pairwise *F*_ST_ values. For full definition of breeds see Table 1.

**Additional file 8: Figure S4.** Cross-validation (CV) error values (a) and number of iterations required to reach convergence (b) calculated through Admixture software runs for *K* values ranging from 2 to 46. The black arrow indicates the *K* = 43 value with the lowest CV score.

**Additional file 9: Figure S5.** Admixture analysis plot in a circular fashion with all values of *K* (number of clusters) ranging from 2 to 43. For full definition of breeds see Table 1.

**Additional file 10: Figure S6.** Optional number of migration events in the complete dataset calculated by using the “*plot_optM*” function in the R package *OptM*. (a) The mean and standard deviation (SD) for the composite likelihood L(m) (left axis, black circles) and proportion of variance explained (right axis, red circles). The 99.8% threshold is that recommended by Pickrell and Pritchard [49], but not visible here because the threshold is still not met at m = 10 edges. (b) The second-order rate of change (Δm) across values of m. The black arrow indicates the peak in Δm at m = 4 edges.

**Additional file 11: Table S5.** Overlapping genomic regions identified with both the Rsb and XP-EHH approaches in the comparison between sheep reared under Mediterranean vs Continental climate.

**Additional file 12.** Description of genes under divergent selection identified by Rsb and XP-EHH approaches.

**Additional file 13: Table S6.** List of identified genomic regions of extended homozygosity (ROH islands).

**Additional file 14.** Description of genes detected by ROH in the overlapping regions with those identified by Rsb and XP-EHH approaches.

**Additional file 15: Figure S7.** Gene network produced by using GeneMANIA. The genes of interest are represented as stripped grey circles, and related genes as plain circles. Co-expressions are displayed as violet lines, physical interactions as red lines, shared pathways as light blue lines, genetic interaction as light green lines, and co-localisations as blue lines.

**Additional file 16: Table S7.** Description of gene functions highlighted in the GeneMANIA network.

## Data Availability

All relevant data are included in the manuscript and its additional files. The datasets used and analysed during the current study are available from the corresponding author upon reasonable request.
