# Supplementary material for: A comprehensive analysis of the genetic diversity and environmental adaptability in worldwide Merino and Merino-derived sheep breeds
Source: Genet Sel Evol. 2023 Apr 3;55:24. doi: 10.1186/s12711-023-00797-z (PMC10069132; doi:10.1186/s12711-023-00797-z)
Supplement: Supplementary file 2 — Additional file 2: Table S2. List of populations excluded from the SNeP software analysis due to a small sample size (number of individuals < 20). [file 12711_2023_797_MOESM2_ESM.docx]

**Additional file 2: Table S2.** List of populations excluded from SNeP software analysis due to small sample size (number of individuals <20).

| **Breed code** | **Breed name** | **Continent** | **Country** |
| --- | --- | --- | --- |
| BDC | Berrichon du Cher | Europe | France |
| FLE | Merinofleischschaf | Europe | Germany |
| MCM | Macarthur Merino | Oceania | Australia |
| MAR | Merino Argentina | South America | Argentina |
| MER | Merino d’Arles | Europe | France |
| SMM | South African Mutton Merino | Africa | South Africa |
| SOV | Soviet Merino | Asia | Russia |
| STA | Stavropol | Asia | Russia |
| TRS | Trimeticcio di Segezia | Europe | Italy |
