# Supplementary figures and images for: A comprehensive analysis of the genetic diversity and environmental adaptability in worldwide Merino and Merino-derived sheep breeds
Source: Genet Sel Evol. 2023 Apr 3;55:24. doi: 10.1186/s12711-023-00797-z (PMC10069132; doi:10.1186/s12711-023-00797-z)

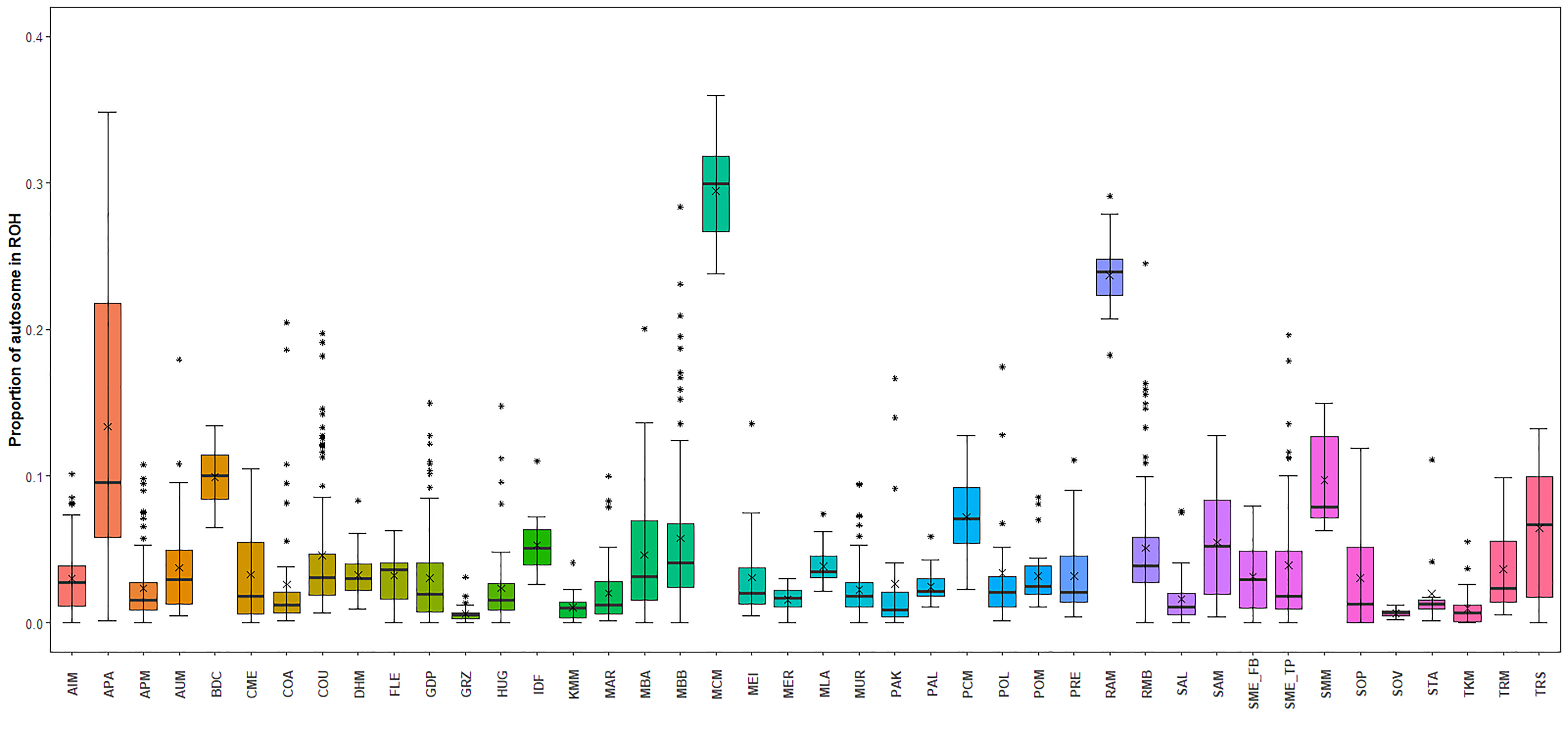

Supplement: Supplementary file 4 — Additional file 4: Figure S1. Distribution of ROH inbreeding coefficients for each Merino and Merino derived breed. [file 12711_2023_797_MOESM4_ESM.tif]

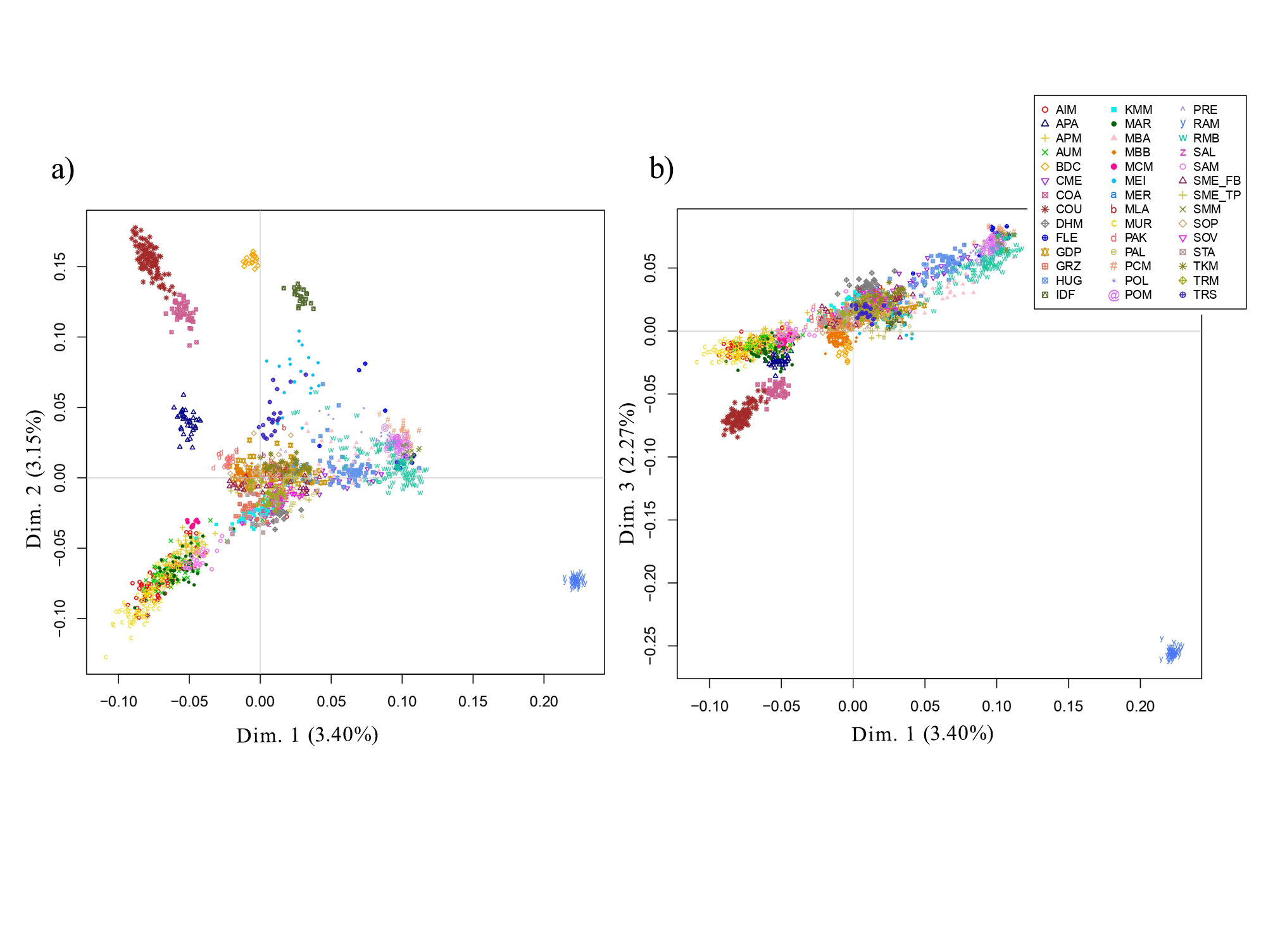

Supplement: Supplementary file 5 — Additional file 5: Figure S2. MDS plots of Dimensions 1 vs 2 (panel a) and 1 vs 3 (panel b). Each point represents a single individual. The correspondence between breeds and symbols is given in the legend box in the upper right corner. For full definition of breeds see Table 1. [file 12711_2023_797_MOESM5_ESM.tif]

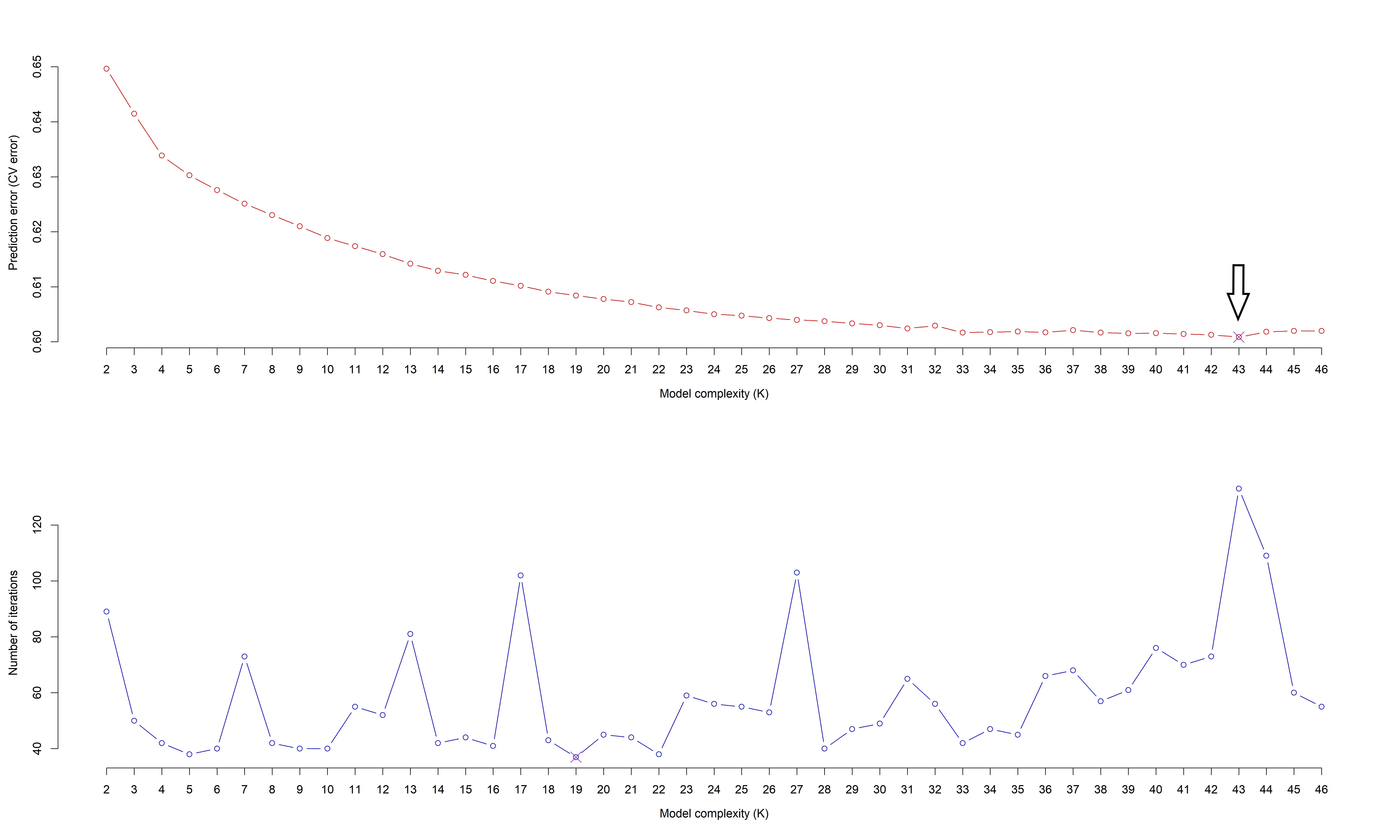

Supplement: Supplementary file 8 — Additional file 8: Figure S4. Cross-validation (CV) error values (a) and number of iterations required to reach convergence (b) calculated through Admixture software runs for K values ranging from 2 to 46. The black arrow indicates the K = 43 value with the lowest CV score. [file 12711_2023_797_MOESM8_ESM.tif]

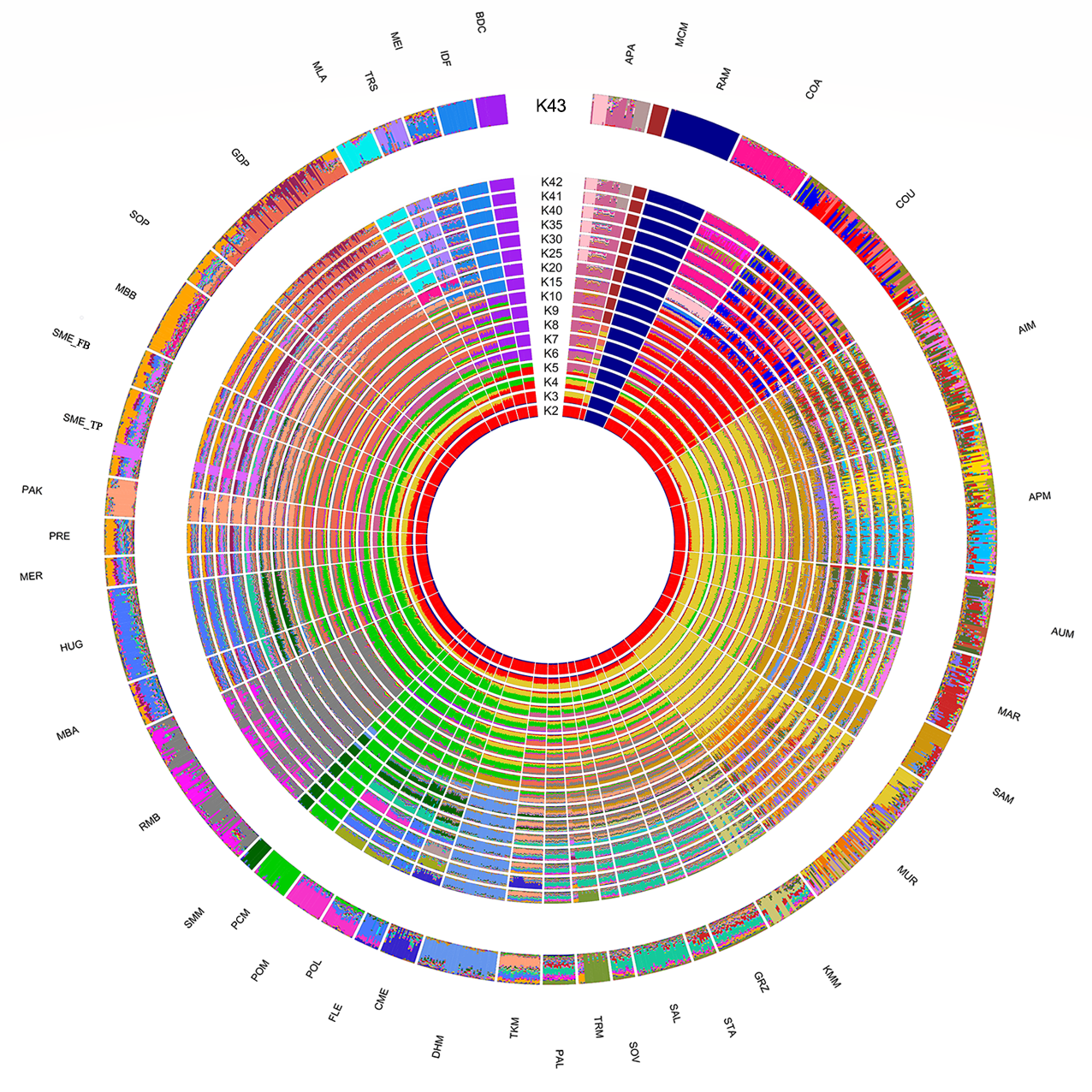

Supplement: Supplementary file 9 — Additional file 9: Figure S5. Admixture analysis plot in a circular fashion with all values of K (number of clusters) ranging from 2 to 43. For full definition of breeds see Table 1. [file 12711_2023_797_MOESM9_ESM.tif]

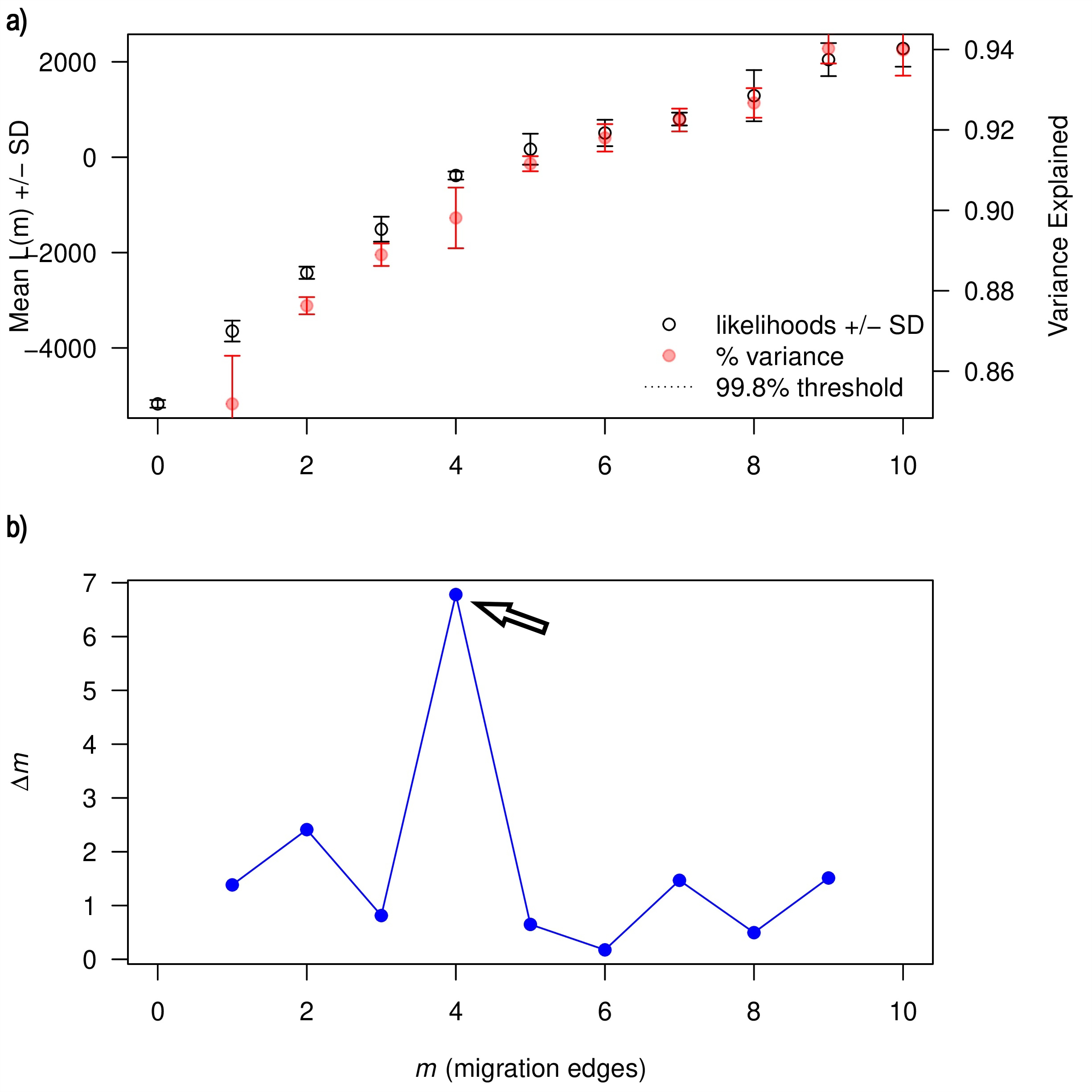

Supplement: Supplementary file 10 — Additional file 10: Figure S6. Optional number of migration events in the complete dataset calculated by using the “plot_optM” function in the R package OptM. (a) The mean and standard deviation (SD) for the composite likelihood L(m) (left axis, black circles) and proportion of variance explained (right axis, red circles). The 99.8% threshold is that recommended by Pickrell and Pritchard [49], but not visible here because the threshold is still not met at m = 10 edges. (b) The second-order rate of change (Δm) across values of m. The black arrow indicates the peak in Δm at m = 4 edges. [file 12711_2023_797_MOESM10_ESM.tif]

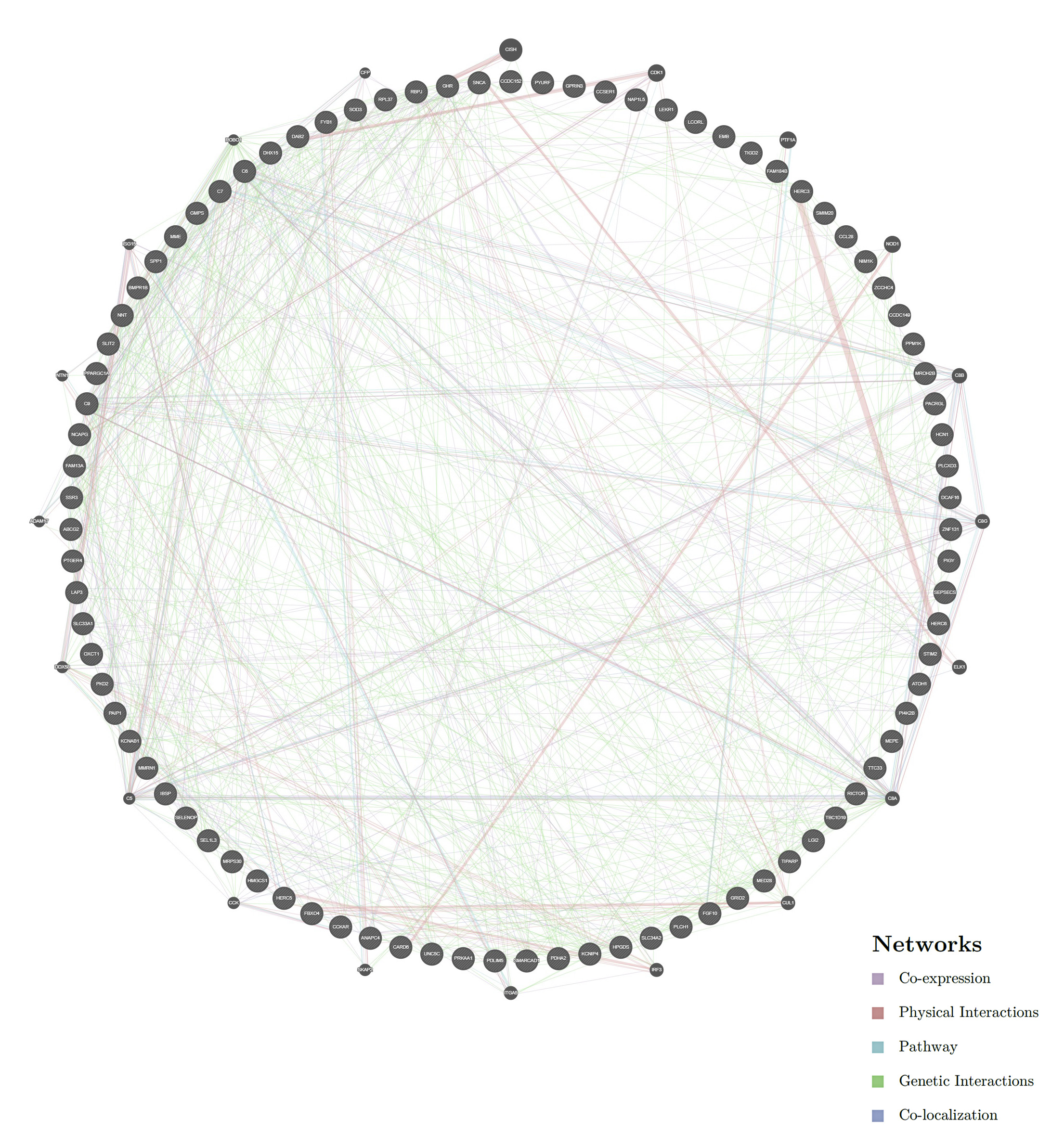

Supplement: Supplementary file 15 — Additional file 15: Figure S7. Gene network produced by using GeneMANIA. The genes of interest are represented as stripped grey circles, and related genes as plain circles. Co-expressions are displayed as violet lines, physical interactions as red lines, shared pathways as light blue lines, genetic interaction as light green lines, and co-localisations as blue lines. [file 12711_2023_797_MOESM15_ESM.jpg]
