# Supplementary material for: A comprehensive analysis of the genetic diversity and environmental adaptability in worldwide Merino and Merino-derived sheep breeds
Source: Genet Sel Evol. 2023 Apr 3;55:24. doi: 10.1186/s12711-023-00797-z (PMC10069132; doi:10.1186/s12711-023-00797-z)
Supplement: Supplementary file 11 — Additional file 11: Table S5. Overlapping genomic regions identified with both the Rsb and XP-EHH approaches in the comparison between sheep reared under Mediterranean vs Continental climate. [file 12711_2023_797_MOESM11_ESM.docx]

**Additional file 11: Table S5.** Overlapping genomic regions identified with both the Rsb and XP-EHH approaches in the comparison between sheep reared under Mediterranean *vs* Continental climate.

| **OAR** | **Start (Mb)** | **End (Mb)** | **Genes** |
| --- | --- | --- | --- |
| 1 | 228.4 | 231.15 | *LEKR1*, *LOC105603748*, *LOC105603748*, *TIPARP*, *LOC105603750*, *SSR3*, *KCNAB1*, *LOC105605210*, *LOC105603753*, *LOC101111596*, *GMPS*, *LOC105603764*, *SLC33A1*, *C1H3orf33*, *LOC105605211*, *PLCH1*, *LOC105603773*, *LOC105603774*, *MME*, *TRNAR-UCU*, *LOC101113188* |
| 6 | 26.5 | 41.65 | *LOC106990267*, *LOC105613656*, *PDHA2*, *UNC5C*, *LOC105615441*, *LOC105609581*, *TRNAK-CUU*, *LOC106991233*, *BMPR1B*, *LOC105615443*, *PDLIM5*, *HPGDS*, *LOC106991234*, *SMARCAD1*, *LOC101102565*, *LOC101116832*, *LOC101102814*, *ATOH1*, *GRID2*, *LOC106991211*, *TRNAS-AGA*, ***CCSER1***, ***TRNAW-CCA***, ***LOC105615447*, *LOC106991208***, *MMRN1*, *SNCA*, *GPRIN3*, *TIGD2*, *FAM13A*, *HERC3*, *NAP1L5*, *PYURF*, *PIGY*, *HERC5*, *HERC6*, *PPM1K*, *ABCG2*, *PKD2*, *SPP1*, *MEPE*, *IBSP*, *LOC101103815*, *TRNAA-CGC*, *LAP3*, *MED28*, *FAM184B*, *LOC105615455*, *NCAPG*, *LOC105608051*, *DCAF16*, *LCORL*, *LOC105615456*, *LOC105608050*, *LOC105608049*, *LOC101104580*, *TRNASTOP-UCA*, *LOC106991224*, ***SLIT2***, ***LOC101122950***, ***PACRGL***, ***KCNIP4***, *LOC105608045*, *LOC106991210*, *TRNAS-GGA*, *LOC105611897*, *LOC106991209*, *LOC105615458*, *LOC101104829*, *TRNAW-CCA* |
| 6 | 43.75 | 46.7 | *PPARGC1A*, *DHX15*, *LOC106991213*, *SOD3*, *CCDC149*, *LOC105608879*, *LGI2*, *SEPSECS*, *PI4K2B*, *ZCCHC4*, *ANAPC4*, *SLC34A2*, *SEL1L3*, *LOC105608876*, *SMIM20*, *TRNAA-UGC*, *TRNAH-AUG*, *LOC105608875*, *RBPJ*, *LOC101107788*, *CCKAR*, *TBC1D19*, *STIM2*, *TRNAL-UAA*, *LOC106991225* |
| 16 | 28.7 | 35.35 | *EMB*, *LOC101114558*, *LOC101114813*, *LOC101115068*, *HCN1*, *LOC101121293*, *MRPS30*, *FGF10*, *NNT*, *LOC105602669*, *PAIP1*, *C16H5orf34*, *C16H5orf28*, *CCL28*, *HMGCS1*, *NIM1K*, *LOC105602558*, *ZNF131*, *LOC105602559*, *LOC101115828*, *LOC101123495*, *LOC105602670*, *LOC105602560*, *SEPP1*, *CCDC152*, *GHR*, *FBXO4*, *C16H5orf51*, *OXCT1*, *TRNAC-GCA*, *TRNAY-GUA*, *PLCXD3*, *C6*, *MROH2B*, *C7*, *CARD6*, *RPL37*, *PRKAA1*, *LOC105602567*, *TTC33*, *PTGER4*, *TRNAE-CUC*, *LOC105602568*, *LOC105602671*, *LOC105602672*, *DAB2*, *C9*, *FYB*, *RICTOR* |

In bold genes also detected by ROH approach.
