# Supplementary material for: A comprehensive analysis of the genetic diversity and environmental adaptability in worldwide Merino and Merino-derived sheep breeds
Source: Genet Sel Evol. 2023 Apr 3;55:24. doi: 10.1186/s12711-023-00797-z (PMC10069132; doi:10.1186/s12711-023-00797-z)
Supplement: Supplementary file 12 — Additional file 12. Description of genes under divergent selection identified by Rsb and XP-EHH approaches. [file 12711_2023_797_MOESM12_ESM.docx]

**Additional file 12.** Description of genes under divergent selection identified by Rsb and XP-EHH approaches.

A total of four genomic regions, located on OAR1, OAR6 and OAR16, were identified by Rsb and XP-EHH approaches:

**OAR1** - A number of known genes, in addition to uncharacterised loci, were located in the region of OAR1 putatively under selection (*LEKR1*, *TIPARP*, *SSR3*, *KCNAB1*, *GMPS*, *SLC33A1*, *PLCH1*, and *MME*). Some of them have been associated with morphological and growth traits (*LEKR1*, *TIPARP*, *SSR3*, *KCNAB1*) and/or with haematological traits related with metabolism and immunity (*LEKR1*, *TIPARP*, *SSR3*, *GMPS*, *SLC33A1*, *PLCH1*, *MME*). In addition, for *SLC33A1*, Gene Ontology points to an involvement in BMP (Bone Morphogenetic Protein) signalling, with the Bone Morphogenetic Protein Receptor 1B (*BMPR1B*) interestingly being among the candidate genes in the large selection signature on OAR6 (26.50 - 41.65 Mb). Moreover, *TIPARP* has been involved in oxygen and glucose deprivation-induced neuronal injury [1].

**OAR6** - The region 26.50 - 41.65 Mb on OAR6 included a large number of characterised and uncharacterised loci (71). Among the characterised genes, *LCORL* clearly appeared as one of the most relevant genes, based on the vast literature on its effects on growth, body size and conformation traits in human [2, 3], sheep [4, 5, 6], goats [7, 8], cattle [9, 10, 11], pigs [12], horses [13, 14, 15], donkeys [16], dogs [17], chickens [18], rabbits [19]. Moreover, a possible role for *LCORL* neighbouring genes cannot be excluded given the frequent pleiotropic nature of the association signals/QTLs detected in the genomic region including *LCORL* [20]. In the ovine species, Liu et al. [21] detected, in the region 35 - 35.2 Mb on OAR6, selection signatures in South African Mutton Merino, a dual-purpose sheep breed whose primary selection goal is meat production, with significantly higher body weight and growth rate compared to the wool-type Merino breeds. In Chinese Merino, by using the OvineSNP50 array, He et al. [22] detected a ROH hotspot in the OAR6 region spanning 35 - 38.67 Mb. In a large population of Australian sheep genotyped with the high-density 600K ovine array, Al Kalaldeh et al. [23] robustly identified significant association signals with parasite resistance in the region between 34.7 and 39.2 Mb on OAR6. Signatures of selection were also identified in the intervals 36.6 - 36.9 and 37.2 - 38.6 Mb of OAR6 by Gutiérrez-Gil et al. [24] using a cross-population extended haplotype homozygosity (XP-EHH) approach performed between Merino (Australian Industry Merino, Australian Merino and Australian Poll Merino) and the coarse-wool Churra population. Also, in Australian Merino sheep, genotyped using the medium-density array, Al-Mamun et al. [25] detected 13 significant SNPs that were associated with birth weight on OAR6, spanning the interval 36.1 and 38.6 Mb.

Other interesting genes in the 26.50 - 41.65 Mb region of OAR6 were: *NCAPG*, encoding for a subunit of the condensing complex, responsible for the condensation and stabilisation of chromosomes during meiosis and mitosis, also identified as a prognostic biomarker of immune infiltration in various cancer types [26]; *BMPR1B*, renowned to be associated with litter size in sheep [27, 28]; *PDLIM5,* encoding for a cytoskeleton-related protein playing an important role in regulating cell proliferation, differentiation and cell fate decision in multiple tissues and cell types; *MED28*, also suggested as participating in cellular signalling to the actin cytoskeleton; *FAM13A*, characterised as an hypoxia-inducible gene [29]; *KCNIP4*, encoding for an integral subunit component of oxygen-sensitive potassium channels [30]; and the members of the HERC family of ubiquitin ligases (*HERC3*, *HERC5*, and *HERC6*), emerging as key components of a wide range of cellular functions, including neurodevelopment, DNA damage repair, cell growth and immune response [31]. Notably, *HERC3* has been involved in UVB-induced cellular senescence *in vitro* [32] and dairy cattle milk somatic cell score [33]. *HERC5* has been involved in antiviral response [34, 35], similarly to *HERC6* [36]. The latter gene product has been also shown to be modulated by interferon [37] and to be associated with feed efficiency in beef heifers [38]. All the three HERC proteins have been detected as having various roles in innate and acquired immune response mechanisms and associated with resistance to gastrointestinal parasites in Australian sheep [23]. Among the genes with a role in immune response, also *SLIT2*, *HPGDS*, *LAP3* (also involved in blood pressure regulation) and *SPP1* (alias *osteopontin*, known to have multiple functions in developmental processes, immunological responses during inflammation and wound healing, and bone mineralization) were identified. Finally, the *MEPE* gene plays a role in bone mineralization and bone mass, encoding for a secreted calcium-binding phosphoprotein.

In the region 43.75 - 46.70 Mb on OAR6, 25 loci were detected. Out of them, the *PPARGC1A* gene, also known as *PGC1alpha*, has repeatedly been proven to have functions in growth, carcass traits, and meat quality in different livestock species [39, 40, 41, 42]. The gene is a transcription coactivator that interacts with multiple transcription factors involved in controlling energy metabolism, lipogenesis, and muscle fiber differentiation [43]. In Chinese sheep, Zhang et al. [44] reported its involvement in the nutrient sensing system by increasing the mitochondrial biogenesis as a consequence of metabolic adaptation to the frequent fluctuations of food availability. Jiao et al. [45] referred that *PPARGC1A* has a regulatory impact on adaptive thermogenesis in cold-exposed fat-tail sheep. For this gene, Sweet-Jones et al. [46] suggested a strong putative selection signature relating to a local adaptation in Welsh upland sheep breeds using sequencing data.

Within the same genomic region, other genes are also included, mainly involved in metabolic pathways and immune response (*PI4K2B*, *ANAPC4*, *RBPJ*, *STIM2*). *PI4K2B* is implicated in the phosphatidylinositol signalling system, promoting a fundamental role in pathogen recognition and activation of innate immunity [47]. Another gene involved in the immunity pathway, *ANAPC4*, also seems to have an important role in cell mediated immunity within the Class I MHC mediated antigen processing and presentation on the cell surface to cytotoxic T lymphocytes. *ANAPC4* has also been found to be associated with carcass traits in Korean beef cattle using a GWAS approach [48]. The *RBPJ* gene is a crucial downstream effector of the “Notch signalling” pathway that is involved in cell proliferation, apoptosis, and the maintenance of stem cells [49]. In the literature, *RBPJ* has also been reported as a key gene for hair follicle differentiation and development in Merino sheep [50]. The *STIM2* gene, encoding the protein stromal interaction molecule 2, is an important regulator of intracellular Ca2+ concentration and myogenesis [51, 52]. Interestingly, *STIM2* is linked to various human cellular disorders and also associated with the development and function of regulatory T cells [53]. Other authors found this gene being associated with muscle growth in Brazilian Nelore beef cattle [54].

**OAR16** - In the region 28.70 - 35.35 Mb on OAR16, 34 known genes were detected under putative selection. Some of them had been associated with immunity (*EMB*, *CCL28*, *SEPP1*, *FBOX4*, *C6*, *C7*, *C9*, *PTGER4*, *FYB1*), metabolic traits (*PRKAA1*), morphogenesis and morphological traits (*FGF10*, *GHR*). Among them, the most noteworthy are *GHR* and *PRKAA1*.

The *GHR* gene, encoding a member of the type I cytokine receptor family, is a transmembrane receptor for growth hormone. In human, *GHR* mutations have been associated with Laron syndrome, a hereditary disorder leading to diminished growth hormone (GH) and phenotypically characterised by short stature [55]. *GHR* is an important growth-related gene affecting meat production and its quality, as well as reproduction traits [56, 57, 58]. For instance, Wang et al. [56] highlighted the importance of *GHR* on meat production and quality of German Mutton Merino sheep. *GHR* has been associated with hypoxic adaptability and growth traits in Tibetan sheep living at high altitude [59, 60]. Recently, it has been recognised as a potential candidate gene for heat tolerance in sheep [61] and goats [62].

The *PRKAA1* gene encodes the alpha1 subunit of the AMP-activated protein kinase (AMPK), which represents the central metabolic switch responsible for maintaining energy homeostasis in response to nutrient and intracellular energy levels alterations [63]. In Tibetan sheep, Zhang et al. [64] identified a set of candidate genes, including *PRKAA1*, directly associated with pathways related to hypoxia responses. *PRKAA1* is also involved in sheep follicular [65] and testicular [66] development.

**References**

1. Shen B, Wang L, Xu Y, Wang H, He S. Knockdown of lncRNA SNHG15 Ameliorates Oxygen and Glucose Deprivation (OGD)-Induced Neuronal Injury via Regulating the miR-9-5p/TIPARP Axis. Biochem Genet. 2022;60:755–69.
2. Lin YJ, Liao WL, Wang CH, Tsai LP, Tang CH, Chen CH, et al. Association of human height-related genetic variants with familial short stature in Han Chinese in Taiwan. Sci Rep. 2017;7:6372.
3. He M, Xu M, Zhang B, Liang J, Chen P, Lee JY, et al. Meta-analysis of genome-wide association studies of adult height in East Asians identifies 17 novel loci. Hum Mol Genet. 2015;24:1791–1800.
4. Posbergh CJ, Huson HJ. All sheeps and sizes: a genetic investigation of mature body size across sheep breeds reveals a polygenic nature. Anim Genet. 2021;52:99–107.
5. Signer‐Hasler H, Burren A, Ammann P, Drögemüller C, Flury C. Runs of homozygosity and signatures of selection: A comparison among eight local Swiss sheep breeds. Anim Genet. 2019;50:512–25.
6. Yurchenko AA, Deniskova TE, Yudin NS, Dotsev AV, Khamiruev TN, Selionova MI, et al. High-density genotyping reveals signatures of selection related to acclimation and economically important traits in 15 local sheep breeds from Russia. BMC Genomics. 2019;20:294.
7. Graber JK, Signer‐Hasler H, Burren A, Drögemüller C. Evaluation of truncating variants in the LCORL gene in relation to body size of goats from Switzerland. Anim Genet. 2022;53:237–9.
8. Saif R, Henkel J, Jagannathan V, Drögemüller C, Flury C, Leeb T. The LCORL locus is under selection in large-sized Pakistani goat breeds. Genes. 2020;11:168.
9. Keogh K, Carthy TR, McClure MC, Waters SM, Kenny DA. Genome-wide association study of economically important traits in Charolais and Limousin beef cows. Animal. 2021;15:100011.
10. Martinez-Castillero M, Then C, Altarriba J, Srihi H, López-Carbonell D, Díaz C, et al. Detection of Genomic Regions with Pleiotropic Effects for Growth and Carcass Quality Traits in the Rubia Gallega Cattle Breed. Animals. 2021;11:1682.
11. Naserkheil M, Mehrban H, Lee D, Park M. Genome-wide Association Study for Carcass Primal Cut Yields Using Single-step Bayesian Approach in Hanwoo Cattle. Front Genet. 2021;12:752424.
12. Schiavo G, Bertolini F, Galimberti G, Bovo S, Dall’Olio S, Costa LN, et al. A machine learning approach for the identification of population-informative markers from high-throughput genotyping data: application to several pig breeds. Animal. 2020;14:223–32.
13. de Faria DA, do Prado Paim T, Dos Santos CA, Paiva SR, Nogueira MB, McManus C (2022). Selection signatures for heat tolerance in Brazilian horse breeds. Mol Genet Genom. 2022;297:449–62.
14. Ablondi M, Dadousis C, Vasini M, Eriksson S, Mikko S, Sabbioni A. Genetic diversity and signatures of selection in a native italian horse breed based on SNP data. Animals. 2020;10:1005.
15. Al Abri MA, Holl HM, Kalla SE, Sutter NB, Brooks SA. Whole genome detection of sequence and structural polymorphism in six diverse horses. PLoS One. 2020;15:e0230899.
16. Shen J, Yu J, Dai X, Li M, Wang G, Chen N, et al. Genomic analyses reveal distinct genetic architectures and selective pressures in Chinese donkeys. J Genet Genomics. 2021;48:737–45.
17. Sheet S, Kim JS, Ko MJ, Kim NY, Lim YJ, Park MR et al. Insight into the Candidate Genes and Enriched Pathways Associated with Height, Length, Length to Height Ratio and Body-Weight of Korean Indigenous Breed, Jindo Dog Using Gene Set Enrichment-Based GWAS Analysis. Animals. 2021;11:3136.
18. Liu J, Zhou J, Li J, Bao H. Identification of candidate genes associated with slaughter traits in F2 chicken population using genome‐wide association study. Anim Genet. 2021;52:532–5.
19. Ballan M, Bovo S, Schiavo G, Schiavitto M, Negrini R, Fontanesi L. Genomic diversity and signatures of selection in meat and fancy rabbit breeds based on high-density marker data. Genet Sel Evol. 2022;54:3.
20. Takasuga A. PLAG1 and NCAPG‐LCORL in livestock. Anim Sci J. 2016;87:159-67.
21. Liu Z, Bai C, Shi L, He Y, Hu M, Sun H, et al. Detection of selection signatures in South African Mutton Merino sheep using whole‐genome sequencing data. Anim Genet. 2022;53:224–9.
22. He S, Di J, Han B, Chen L, Liu M, Li W. Genome-wide scan for runs of homozygosity identifies candidate genes related to economically important traits in Chinese Merino. Animals. 2020;10:524.
23. Al Kalaldeh M, Gibson J, Lee SH, Gondro C, Van Der Werf JH. Detection of genomic regions underlying resistance to gastrointestinal parasites in Australian sheep. Genet Sel Evol. 2019;51:37.
24. Gutiérrez-Gil B, Esteban-Blanco C, Wiener P, Chitneedi PK, Suarez-Vega A, Arranz JJ. High-resolution analysis of selection sweeps identified between fine-wool Merino and coarse-wool Churra sheep breeds. Genet Sel Evol. 2017;49:81.
25. Al-Mamun HA, Kwan P, Clark SA, Ferdosi MH, Tellam R, Gondro C. Genome-wide association study of body weight in Australian Merino sheep reveals an orthologous region on OAR6 to human and bovine genomic regions affecting height and weight. Genet Sel Evol. 2015;47:66.
26. Zhou Y, Fan Y, Mao Y, Lou M, Liu X, Yuan K, et al. NCAPG is a prognostic biomarker of immune infiltration in non-small-cell lung cancer. Biomark Med. 2022;16:523–35.
27. Yang Z, Yang X, Liu G, Deng M, Sun B, Guo Y, et al. Polymorphisms in BMPR-IB gene and their association with litter size trait in Chinese Hu sheep. Anim Biotechnol. 2022;33:250–9.
28. Gholizadeh M, Esmaeili-Fard SM. Meta-analysis of genome-wide association studies for litter size in sheep. Theriogenology. 2022;180:103–12.
29. Liang C, Wang G, Raza SHA, Wang X, Li B, Zhang W, Zan L. FAM13A promotes proliferation of bovine preadipocytes by targeting Hypoxia-Inducible factor-1 signaling pathway. Adipocyte. 2021;10:546–57.
30. Patel AJ, Honoré E. Properties and modulation of mammalian 2P domain K+ channels. Trends Neurosci. 2001;24:339–46.
31. Sánchez-Tena S, Cubillos-Rojas M, Schneider T, Rosa JL. Functional and pathological relevance of HERC family proteins: a decade later. Cell Mol Life Sci. 2016;73:1955–68.
32. Zheng X, Chen L, Jin S, Xiong L, Chen H, Hu K, et al. Ultraviolet B irradiation up‐regulates MM1 and induces photoageing of the epidermis. Photodermatol Photoimmunol Photomed. 2021;37:395–403.
33. Ilie DE, Mizeranschi AE, Mihali CV, Neamț RI, Goilean GV, Georgescu OI, et al. Genome-Wide Association Studies for Milk Somatic Cell Score in Romanian Dairy Cattle. Genes. 2021;12:1495.
34. Hasankhani A, Bahrami A, Sheybani N, Fatehi F, Abadeh R, Farahani HGM, et al. Integrated Network Analysis to Identify Key Modules and Potential Hub Genes Involved in Bovine Respiratory Disease: A Systems Biology Approach. Front Genet. 2021;12:753839.
35. Wang K, Thomas C, Zhang S, Wathes DC, Cheng Z. Comparison of the Ability of High and Low Virulence Strains of Non-cytopathic Bovine Viral Diarrhea Virus-1 to Modulate Expression of Interferon Tau Stimulated Genes in Bovine Endometrium. Front Vet Sci. 2021;8:659330.
36. Suen WW, Imoda M, Thomas AW, Nasir NN, Tearnsing N, Wang W, et al. An acute stress model in New Zealand white rabbits exhibits altered immune response to infection with West Nile Virus. Pathogens. 2019;8:195.
37. Forde N, Duffy GB, McGettigan PA, Browne JA, Mehta JP, Kelly AK, et al. Evidence for an early endometrial response to pregnancy in cattle: both dependent upon and independent of interferon tau. Physiol Genomics. 2012;44:799–810.
38. Paradis F, Yue S, Grant JR, Stothard P, Basarab JA, Fitzsimmons C. Transcriptomic analysis by RNA sequencing reveals that hepatic interferon-induced genes may be associated with feed efficiency in beef heifers. J Anim Sci. 2015;93:3331–41.
39. Chen P, Zhao H, Wu M, He S, Yuan T, Yi X, et al. A novel 17 bp InDel polymorphism within the PPARGC1A gene is significantly associated with growth traits in sheep. Anim Biotechnol. 2022;33:312–20.
40. Liao Y, Wang Z, Glória LS, Zhang K, Zhang C, Yang R, et al. Genome-wide association studies for growth curves in meat rabbits through the single-step nonlinear mixed model. Front Genet. 2021;12:750939.
41. Fernández-Barroso MÁ, Caraballo C, Silió L, Rodríguez C, Nuñez Y, Sánchez-Esquiliche F, et al. Differences in the loin tenderness of Iberian Pigs explained through dissimilarities in their transcriptome expression profile. Animals. 2020;10:1715.
42. Armstrong E, Ciappesoni G, Iriarte W, Da Silva C, Macedo F, Navajas EA, et al. Novel genetic polymorphisms associated with carcass traits in grazing Texel sheep. Meat Sci. 2018;145:202–8.
43. Soria LA, Corva PM, Sica AB, Villarreal EL, Melucci LM, Mezzadra CA, et al. Association of a novel polymorphism in the bovine PPARGC1A gene with growth, slaughter and meat quality traits in Brangus steers. Mol Cell Probes. 2009;23:304–8.
44. Zhang GM, Zhang TT, Jin YH, Liu JL, Guo YX, Fan YX, et al. Effect of caloric restriction and subsequent re-alimentation on oxidative stress in the liver of Hu sheep ram lambs. Anim Feed Sci Technol. 2018;237:68–77.
45. Jiao D, Ji KX, Liu H, Wang WQ, Wu X, Zhou J, et al. Transcriptome analysis reveals genes involved in thermogenesis in two cold-exposed sheep breeds. Genes. 2021;12:375.
46. Sweet-Jones J, Lenis VP, Yurchenko AA, Yudin NS, Swain M, Larkin DM. Genotyping and Whole-Genome Resequencing of Welsh Sheep Breeds Reveal Candidate Genes and Variants for Adaptation to Local Environment and Socioeconomic Traits. Front Genet. 2021;12:612492.
47. Bilodeau P, Jacobsen D, Law-Vinh D, Lee JM. Phosphatidylinositol 4-kinase III beta regulates cell shape, migration, and focal adhesion number. Mol Biol Cell. 2020;31:1904–16.
48. Srikanth K, Lee SH, Chung KY, Park JE, Jang GW, Park MR, et al. A gene-set enrichment and protein–protein interaction network-based GWAS with regulatory SNPs identifies candidate genes and pathways associated with carcass traits in Hanwoo cattle. Genes. 2020;11:316.
49. Lake RJ, Tsai PF, Choi I, Won KJ, Fan HY. RBPJ, the major transcriptional effector of Notch signaling, remains associated with chromatin throughout mitosis, suggesting a role in mitotic bookmarking. PLoS Genet. 2014;10:e1004204.
50. Zhao B, Luo H, He J, Huang X, Chen S, Fu X, et al. Comprehensive transcriptome and methylome analysis delineates the biological basis of hair follicle development and wool-related traits in Merino sheep. BMC Biol. 2021b;19:197.
51. Berna-Erro A, Braun A, Kraft R, Kleinschnitz C, Schuhmann MK, Stegner D, et al. STIM2 regulates capacitive Ca2+ entry in neurons and plays a key role in hypoxic neuronal cell death. Sci Signal. 2009; 2:ra67.
52. Kim KM, Rana A, Park CY. Orai1 inhibitor STIM2β regulates myogenesis by controlling SOCE dependent transcriptional factors. Sci Rep. 2019;9:10794.
53. Oh-Hora M, Yamashita M, Hogan PG, Sharma S, Lamperti E, Chung W, et al. Dual functions for the endoplasmic reticulum calcium sensors STIM1 and STIM2 in T cell activation and tolerance. Nat Immunol. 2008;9:432–43.
54. Mudadu MA, Porto-Neto LR, Mokry FB, Tizioto PC, Oliveira PS, Tullio RR, et al. Genomic structure and marker-derived gene networks for growth and meat quality traits of Brazilian Nelore beef cattle. BMC Genomics. 2016;17:235.
55. Hinrichs A, Renner S, Bidlingmaier M, Kopchick JJ, Wolf E. MECHANISMS IN ENDOCRINOLOGY: Transient juvenile hypoglycemia in growth hormone receptor deficiency–mechanistic insights from Laron syndrome and tailored animal models. Eur J Endocrinol. 2021;185:R35–R47.
56. Wang H, Zhang L, Cao J, Wu M, Ma X, Liu Z, et al. Genome-wide specific selection in three domestic sheep breeds. PLoS One, 2015;10:e0128688.
57. Gorlov IF, Kolosov YA, Shirokova NV, Getmantseva LV, Slozhenkina MI, Mosolova NI, et al. Association of the growth hormone gene polymorphism with growth traits in Salsk sheep breed. Small Rumin Res. 2017;150:11–4.
58. Yang Q, Yan H, Li J, Xu H, Wang K, Zhu H, et al. A novel 14-bp duplicated deletion within goat GHR gene is significantly associated with growth traits and litter size. Anim Genet. 2017;48:499–500.
59. Li LL, Ma SK, Peng W, Fang YG, Duo HR, Fu HY, et al. Genetic diversity and population structure of Tibetan sheep breeds determined by whole genome resequencing. Trop Anim Health Prod. 2021;53:174.
60. Han YC, Sun YG, Li Q. Growth hormone polymorphisms and growth traits in Chinese Tibetan sheep Ovis aries. Genet Mol Res. 2016;15.
61. Sejian V, Bagath M, Krishnan G, Rashamol VP, Pragna P, Devaraj C, et al. Genes for resilience to heat stress in small ruminants: A review. Small Rumin Res. 2019;173:42–53.
62. Angel SP, Bagath M, Sejian V, Krishnan G, Bhatta R. Expression patterns of candidate genes reflecting the growth performance of goats subjected to heat stress. Mol Biol Rep. 2018;45:2847–56.
63. Hardie DG, Ross FA, Hawley SA. AMPK: a nutrient and energy sensor that maintains energy homeostasis. Nat Rev Mol Cell Biol. 2012;13:251–62.
64. Zhang Y, Xue X, Liu Y, Abied A, Ding Y, Zhao S, et al. Genome-wide comparative analyses reveal selection signatures underlying adaptation and production in Tibetan and Poll Dorset sheep. Sci Rep. 2021;11:2466.
65. Foroughinia G, Fazileh A, Eghbalsaied S. Expression of genes involved in BMP and estrogen signaling and AMPK production can be important factors affecting total number of antral follicles in ewes. Theriogenology. 2017;91:36–43.
66. Pang J, Li F, Feng X, Yang H, Han L, Fan Y, et al. Influences of different dietary energy level on sheep testicular development associated with AMPK/ULK1/autophagy pathway. Theriogenology. 2018;108:362–70.
