# Supplementary material for: A comprehensive analysis of the genetic diversity and environmental adaptability in worldwide Merino and Merino-derived sheep breeds
Source: Genet Sel Evol. 2023 Apr 3;55:24. doi: 10.1186/s12711-023-00797-z (PMC10069132; doi:10.1186/s12711-023-00797-z)
Supplement: Supplementary file 13 — Additional file 13: Table S6. List of identified genomic regions of extended homozygosity (ROH islands). [file 12711_2023_797_MOESM13_ESM.docx]

**Additional file 13: Table S6.** List of genomic regions of extended homozygosity (ROH islands) identified.

| Group | OAR | Number of SNPs | Number of genes | Start (Mb) | End (Mb) |
| --- | --- | --- | --- | --- | --- |
| Mediterranean climate | 5 | 5 | 1 | 67.29 | 67.60 |
|  | 6 | 12 | 4 | 38.96 | 40.15 |
|  | 6 | 14 | 0 | 42.36 | 43.06 |
|  | 10 | 24 | 14 | 27.37 | 29.16 |
|  | 12 | 16 | 13 | 46.49 | 47.50 |
| Continental climate | 6 | 25 | 4 | 32.38 | 34.56 |
