# Supplementary material for: A comprehensive analysis of the genetic diversity and environmental adaptability in worldwide Merino and Merino-derived sheep breeds
Source: Genet Sel Evol. 2023 Apr 3;55:24. doi: 10.1186/s12711-023-00797-z (PMC10069132; doi:10.1186/s12711-023-00797-z)
Supplement: Supplementary file 14 — Additional file 14. Description of genes detected by ROH in the overlapping regions with those identified by Rsb and XP-EHH approaches. [file 12711_2023_797_MOESM14_ESM.docx]

**Additional file 14.** Description of genes detected by ROH in the overlapping regions with those identified by Rsb and XP-EHH approaches.

ROH island approach identified two regions on OAR6, characterised by a high frequency of ROH occurrence. These regions included eight genes (*SLIT2*, *LOC101122950*, *PACRGL*, *KCNIP4;* *CCSER1* - alias *FAM190A*, *TRNAW-CCA*, *LOC105615447*, and *LOC106991208).* Following*,* the main biological functions are reported for *SLIT2*, *CCSER1* (alias *FAM190A*), *PACRGL*, and *KCNIP4*.

***SLIT2*** - This gene encodes a member of the slit family of secreted glycoproteins, which are ligands for the Robo (Roundabout) family of immunoglobulin receptors. Slit proteins play highly conserved roles in axon guidance and neuronal migration and may also have functions during other cell migration processes including leukocyte migration [1]. During inflammation, leukocytes must migrate from the blood circulation into the interstitium of the involved tissue. Inflammatory cells respond to an orchestra of signals and eventually arrive at the appropriate site. While several studies have established the importance of positive regulators in controlling leukocyte chemotaxis, there has been only limited evidence for the existence of endogenous inhibitory regulators of this process [2].

*SLIT2* has been also shown to selectively impair neutrophil migration and inhibit neutrophil chemotaxis. In the model of renal ischemia-reperfusion injury, *SLIT2* blocked the capture and firm adhesion of human neutrophils to the inflamed vascular endothelial barrier. Another observation in a mouse model that *SLIT2* significantly reduces the recruitment of neutrophils to the site of inflammation is in accordance with other inflammation models, including glomerulonephritis-associated kidney injury, global cerebral ischemia, and skin sensitization to allergens. One study found that SLIT/ROBO4 strengthens the vascular barrier and diminishes deleterious aspects of the host's response to the pathogen-induced cytokine reaction. Collectively, these evidence shows that Slit/Robo inhibits chemotaxis of leukocytes toward chemoattractants and may have a therapeutic role as an inhibitor of inflammatory cell infiltration [3].

In addition, *SLIT2* has been shown to represent a thyrotropin-inducible factor within the thyroid-associated ophthalmopathy that can modulate the inflammatory phenotype of CD34+ orbital fibroblasts, therefore determining the activity and severity of the disease [4].

Evidence that SLIT2/ROBO2 signaling mediates the pathogenesis of hepatic fibrosis (a characteristic consequence of multiple chronic liver injuries, including metabolic disorders, toxins, viral hepatitis, steatohepatitis, and autoimmune diseases) has been provided [5].

SLIT2/ROBO1 signaling has been shown to inhibit macropinocytosis in vitro and in vivo by inducing cytoskeletal changes in macrophages. Several growth factors and inflammatory stimuli are known to induce macropinocytosis. Macropinocytosis is essential for myeloid cells to survey their environment [6].

Netrin-1, which is a member of the SLIT2/ROBO1 signaling, has been shown to be an effective anti-inflammatory agent and to protect against acute lung injury in sepsis rats through its anti-inflammation effect [7], as well as to augment CD4(+) T cell chemokinesis and promote cellular infiltration in association with acute inflammation in vivo [8].

*SLIT2* has been suggested as a new treatment strategy to arrest chronic injury progression after ischemic and obstructive renal insults by attenuating inflammation and inhibiting renal fibrosis [9].

*SLIT2* has also been suggested to be beneficial to reduce surgical brain injury-induced neuroinflammation [10]. Limiting the disruptive effects of proinflammatory mediators on the vasculature is vital to limit the injury of host. During influenza A viruses (IAV) infection, cytokines produced in host inflammatory response led to vascular leak and accumulation of protein-rich edema fluid in the alveolar space. Binding with ROBO4, *SLIT2* enhances vascular barrier function against multiple cytokines. Thus, this strengthened barrier can protect host from the lethal effects of inflammatory response induced by influenza infection [11].

Slit2N, a ligand for the Roundabout (Robo) receptors, has been shown to block HIV-1-induced signaling and inhibited cell-to-cell transmission of HIV-1 in a Robo1-dependent manner, and inhibited HIV-1 replication through mechanisms involving modulation of cytoskeletal dynamics [12, 13].

In the canine species, a ~ 20kb long haplotype on the chromosome 28, significantly associated to IgA levels in Shar-Pei, was positioned within the first intron of the gene *SLIT1*, with Immunoglobulin A deficiency being the most common primary immune deficiency disorder in dogs [14].

Expression of *SLIT2* in skin has been reported to be upregulated at both 4 hours and 48 hours following hapten sensitization suggesting *SLIT2* can have a role both early and late in an inflammatory response [15].

In chicken, in a GWAS study, a region positioned at 100 Mb from the proximal end of chromosome 1, including the ROBO1 and ROBO2 genes, encoding integral membrane protein receptors for the SLIT-family, had a strong effect on the antibody response to the Newcastle disease virus [16].

Hypoxia significantly increased both mRNA and protein levels of *SLIT2* in BeWo cells and of *SLIT3*, ROBO1, and ROBB4 in HUVEC cell lines [17].

***CCSER1*** **-** The coiled-coil serine-rich protein 1 (*CCSER1*) gene is reported to be related to economic traits in livestock. It has been detected in putative selection signatures detected in the Jiaxian Red cattle contrasted with commercial beef breeds, suggesting a role in growth and feed efficiency [18]. SNPs genotypes within *CCSER1* showed significant over-dominance effects in Angus-Hereford crossbred cattle [19]. It encompasses a QTL associated with weight after slaughter in Hanwoo cattle [20]. It has been found associated to backfat thickness in a genome-wide association study in Chuying-black pigs [21]. In this gene, CNVs have been reported to be associated with growth traits in goats. It has been found among the genes included in selection signatures in the genomes of 15 local Russian sheep breeds, where it is suggested to have a possible role in growth and feed intake [22], and in Chinese Mongolian fat-tailed sheep, where it is suggested to have a role in marbling [23]. It has been proposed, via in vitro studies, to function as a regulator or structural component required for normal mitosis [24].

***PACRGL*** *-* Parkin Coregulated Like is a Protein Coding gene. Gene Ontology (GO) annotations related to this gene include binding. There is a dramatic lack of knowledge about the function of this gene. It has been detected in a selection signature in South African Mutton Merino sheep [25], where it is suggested to be related with fat deposition traits, and in a QTL affecting growth and muscle mass in chicken [26]. An important paralog of this gene is *PACRG*. In *Ovis aries*, it is located on chromosome 8. It was identified in 2003 while studying mutations at the nearby *Parkin* gene in humans where it represents the predominant genetic cause of early-onset and autosomal recessive juvenile parkinsonism [27]. *Parkin* is a multi-domain protein with ubiquitin-protein E3 ligase activity that has a role in the proteasome-mediated degradation of target substrates. At that time, *PACRG* was suggested via in silico prediction to have a potential link to the ubiquitin/proteasome system, evidence experimentally confirmed later on [28]. It was shown to form a large molecular chaperone complex containing heat shock proteins 70 and 90 and chaperonin components [29]. Via positional cloning, *PARK2/PACRG* were identified in humans to represent a worldwide risk factor for leprosy, a chronic infectious disease caused by *Mycobacterium leprae* [30]. It has also been included among the susceptibility candidate genes for tuberculosis, a common infectious disease caused by *M. tuberculosis* [31]. It was suggested to play a role in susceptibility to the intracellular pathogens *S. typhi* and *S. paratyphi* [32]. It was detected among the candidate genes in a genome-wide association studies on Holstein cow’s milk samples collected after an intramammary experimental challenge with *S. uberis* [33]. *PACRG* was shown to play a role in tumor necrosis factor (*TNF*) signaling. Similarly to Parkin, *PACRG* promoted nuclear factor κB (NF-κB) activation in response to *TNF*. This function of *PACRG* in positively regulating TNF signaling may help to explain the association of *PACRG* and *PRKN* polymorphisms with an increased susceptibility to intracellular pathogens [34]. In patients with autoimmune thyroid disease compared to controls, the gene was shown to display an expression that was anti-correlated with that of miRNAs; moreover, the miRNA/mRNAs differential expression was associated with cilia organization [35]. It was also demonstrated that, together with *PARK2*, *PACRG* is epigenetically regulated in human leukemia, suggesting that abnormal methylation and regulation of *PARK2* and *PACRG* may play a role in the pathogenesis and development of this hematological neoplasm [36]. Since male mice homozygous for the *quaking viable* mutation, lacking *PACRG,* are sterile, *PACRG*, which is expressed at high levels in the testis, has been suggested to play a key role in spermatogenesis [37, 38]. In 2005, to elucidate the involvement of *PACRG* in sterility, the gene was demonstrated to function within the axoneme, where *PACRG* would act to maintain functional stability of the axonemal outer doublets of both motile and sensory cilia and flagella [38]. Co-sedimentation experiments revealed that *PACRG* directly binds to microtubules and alpha/beta-tubulin heterodimers with high affinity. Microscopic studies showed that *PACRG* bundles microtubules and forms branched aggregates with unpolymerised tubulin dimers [39]. It has been shown to be a component of the ependymal cilia and possibly be involved in ciliopathies [40]. Cell sensory/signaling functions have also been suggested for *PACRG* [41]. Variation in the promoter of *PACRG* was demonstrated to be a risk factor associated with azoospermia in humans [42]. It was shown to associate with MEIG1, a protein whose inactivating mutations were shown to generate sterile mice as a result of impaired spermatogenesis at the stage of elongation and condensation, overall suggesting a critical role for the MEIG1/PARCG partnership in manchette structure and function and the control of spermiogenesis [43]. In cattle, it was found associated with testis maturation [44]. In humans, variants at the *PACRG* gene were found in infertile men [45].

***KCNIP4*** *-* This gene encodes a member of the family of voltage-gated potassium (Kv) channel-interacting proteins (KCNIPs), which belong to the recoverin branch of the EF-hand superfamily [46]. Members of the *KCNIP* family are small calcium binding proteins. *KCNIP4* has been identified as a β-catenin/Nurr1 target that modulates Wnt signaling by interacting with the presenilin complex [46, 47]. *KCNIP4* has been suggested as a candidate gene for renal cell cancer [48], lung cancer, pancreatic cancer [49], childhood acute lymphoblastic leukemia [50], ACE inhibitor-induced cough [51]. An alternative *KCNIP4* isoform has been described, that cannot interact with the γ-secretase complex, resulting in modification of γ-secretase activity, amyloid precursor protein processing, and increased secretion of β-amyloid enriched in the more toxic Aβ x-42 species. Notably, synthesis of the variant *KCNIP4* isoform is also detrimental to brain physiology, as it results in the concomitant blockade of the fast kinetics of potassium channels. This alternative splicing shift is observed at high frequency in tissue samples from Alzheimer's disease patients [52]. It has also suggested as a candidate gene for attention-deficit/hyperactivity disorder, a neurodevelopmental disorder in children with striking persistence into adulthood and a high co-morbidity with other psychiatric disorders, including personality disorders [53]. In dogs, it has been suggested a candidate gene for cerebellar ataxia [54]. It was identified as a potential target gene in pathogenesis of autism [55] and epilepsy [56]. It was also identified as associated with asthma [57], a common chronic respiratory disease characterised by airway hyperresponsiveness and chronic kidney disease [58]. Together with miR-3068-3p, it has been suggested to serve as a novel target for the treatment of ischemic stroke [59]. It was shown, through a whole-genome cardiac DNA methylation fingerprint and gene expression analysis in chronic Chagas disease cardiomyopathy, that methylation modified expression of key genes, among which *KCNIP4* gene [60]. SNPs in *KCNIP3* were found to be closely associated with growth traits in a genome-wide association study carried out in Red Angus cattle [61], with body weigh in Chinese Yancheng chickens [62], Korean Native Chickens [63] and a chicken F2 resource population [64], and with reproductive traits in a local Chinese chicken [65].

**References**

1. https://www.genecards.org/cgi-bin/carddisp.pl?gene=SLIT2.
2. Kanellis J, Garcia GE, Li P, Parra G, Wilson CB, Rao Y, et al. Modulation of inflammation by slit protein in vivo in experimental crescentic glomerulonephritis. Am J Clin Pathol. 2004;165:341–52.
3. Tong M, Jun T, Nie Y, Hao J, Fan D. The role of the Slit/Robo signaling pathway. J Cancer. 2019;10:2694.
4. Fernando R, Grisolia ABD, Lu Y, Atkins S, Smith TJ Slit2 modulates the inflammatory phenotype of orbit-infiltrating fibrocytes in Graves’ disease. J Immunol. 2018;200:3942–9.
5. Zeng Z, Wu Y, Cao Y, Yuan Z, Zhang Y, Zhang DY, et al. Slit2-Robo2 signaling modulates the fibrogenic activity and migration of hepatic stellate cells. Life Sci. 2018;203:39–47.
6. Bhosle VK, Mukherjee T, Huang YW, Patel S, Pang BWF, Liu GY, et al. SLIT2/ROBO1-signaling inhibits macropinocytosis by opposing cortical cytoskeletal remodeling. Nat Commun. 2020;11:1–17.
7. Liu J, Du J, Cheng X, Zhang X, Li Y, Fu X, et al. Effect of netrin-1 anti-inflammatory factor on acute lung injury in sepsis rats. Med Sci Monit. 2019;25:7928–35.
8. Boneschansker L, Nakayama H, Eisenga M, Wedel J, Klagsbrun M, Irimia D, et al. Netrin-1 augments chemokinesis in CD4+ T cells in vitro and elicits a proinflammatory response in vivo. J Immunol. 2016;197:1389–98.
9. Yuen DA, Huang YW, Liu GY, Patel S, Fang F, Zhou J, et al. Recombinant N–Terminal Slit2 Inhibits TGF-β–Induced Fibroblast Activation and Renal Fibrosis. J Am Soc Nephrol. 2016;27:2609–15.
10. Sherchan P, Huang L, Wang Y, Akyol O, Tang J, Zhang JH. Recombinant Slit2 attenuates neuroinflammation after surgical brain injury by inhibiting peripheral immune cell infiltration via Robo1-srGAP1 pathway in a rat model. Neurobiol Dis. 2016;85:164–73.
11. Yu M, Wang Q, Qi W, Zhang K, Liu J, Tao P, et al. Expression of inflammation-related genes in the lung of BALB/c mice response to H7N9 influenza A virus with different pathogenicity. Med Microbiol Immunol. 2016;205:501–9.
12. Shrivastava A, Prasad A, Kuzontkoski PM, Yu J, Groopman JE. Slit2N inhibits transmission of HIV-1 from dendritic cells to T-cells by modulating novel cytoskeletal elements. Sci Rep. 2015;5:1–14.
13. Anand AR, Zhao H, Nagaraja T, Robinson LA, Ganju RK. N-terminal Slit2 inhibits HIV-1 replication by regulating the actin cytoskeleton. Retrovirology. 2013;10:1–16.
14. Olsson M, Tengvall K, Frankowiack M, Kierczak M, Bergvall K, Axelsson E, et al. Genome-wide analyses suggest mechanisms involving early B-cell development in canine IgA deficiency. PLoS One. 2015;10:e0133844.
15. Coleman DJ, Garcia G, Hyter S, Jang HS, Chagani S, Liang X, et al. Retinoid-X-receptors (α/β) in melanocytes modulate innate immune responses and differentially regulate cell survival following UV irradiation. PLoS Genet. 2014;10:e1004321.
16. Luo C, Qu H, Ma J, Wang J, Li C, Yang C, et al. Genome-wide association study of antibody response to Newcastle disease virus in chicken. BMC Genet. 2013;14:1–9.
17. Liao WX, Laurent LC, Agent S, Hodges J, Chen DB. Human placental expression of SLIT/ROBO signaling cues: effects of preeclampsia and hypoxia. Biol Reprod. 2012;86:111.
18. Xia X, Zhang S, Zhang H, Zhang Z, Chen N, Li Z, et al. Assessing genomic diversity and signatures of selection in Jiaxian Red cattle using whole-genome sequencing data. BMC Genomics. 2021;22:1–11.
19. Abo-Ismail MK, Lansink N, Akanno E, Karisa BK, Crowley JJ, Moore SS, et al. Development and validation of a small SNP panel for feed efficiency in beef cattle. J Anim Sci. 2018;96:375–97.
20. Kim J, Li Y, Lee J, Lee Y. Application of linkage disequilibrium mapping methods to detect QTL for carcass quality on chromosome 6 using a high density SNP map in Hanwoo. Asian-Aust J Anim Sci. 2011;24:457–62.
21. Xue Y, Li C, Duan D, Wang M, Han X, Wang K, et al. Genome‐wide association studies for growth‐related traits in a crossbreed pig population. Anim Genet. 2021;52:217–22.
22. Yurchenko AA, Deniskova TE, Yudin NS, Dotsev AV, Khamiruev TN, Selionova MI, et al. High-density genotyping reveals signatures of selection related to acclimation and economically important traits in 15 local sheep breeds from Russia. BMC Genomics. 2019;20:1–19.
23. Wang H, Zhang L, Cao J, Wu M, Ma X, Liu Z, et al. Genome-wide specific selection in three domestic sheep breeds. PloS One. 2015;10:e0128688.
24. Patel K, Scrimieri F, Ghosh S, Zhong J, Kim MS, Ren YR, et al. FAM190A deficiency creates a cell division defect. Am J Pathol. 2013;183:296–303.
25. Liu Z, Bai C, Shi L, He Y, Hu M, Sun H, et al. (2022). Detection of selection signatures in South African Mutton Merino sheep using whole‐genome sequencing data. Anim Genet. 2022;53:224–9.
26. Lyu S, Arends D, Nassar MK, Brockmann GA. Fine mapping of a distal chromosome 4 QTL affecting growth and muscle mass in a chicken advanced intercross line. Anim Genet. 2017;48:295–302.
27. West AB, Lockhart PJ, O'Farell C, Farrer MJ. Identification of a novel gene linked to parkin via a bi-directional promoter. J Mol Biol. 2003;326:11–9.
28. Taylor JM, Brody KM, Lockhart PJ. Parkin co-regulated gene is involved in aggresome formation and autophagy in response to proteasomal impairment. Exp Cell Res. 2012;318:2059–70.
29. Taylor JM, Song YJC, Huang Y, Farrer MJ, Delatycki MB, Halliday GM, et al. Parkin Co-regulated Gene (PACRG) is regulated by the ubiquitin–proteasomal system and is present in the pathological features of parkinsonian diseases. Neurobiol Dis. 2007;27:238–47.
30. Mira MT, Alcais A, Van Thuc N, Moraes MO, Di Flumeri C, Hong Thai VU, et al. Susceptibility to leprosy is associated with PARK2 and PACRG. Nature. 2004;427:636–40.
31. Bragina EY, Tiys ES, Rudko AA, Ivanisenko VA, Freidin MB. Novel tuberculosis susceptibility candidate genes revealed by the reconstruction and analysis of associative networks. Infect Genet Evol. 2016;46:118–23.
32. Ali S, Vollaard AM, Widjaja S, Surjadi C, Van De Vosse E, Van Dissel JT. PARK2/PACRG polymorphisms and susceptibility to typhoid and paratyphoid fever. Clin Exp Immunol. 2006;144:425–31.
33. Siebert L, Staton ME, Headrick S, Lewis M, Gillespie B, Young C, el al. Genome-wide association study identifies loci associated with milk leukocyte phenotypes following experimental challenge with Streptococcus uberis. Immunogenetics. 2018;70:553–62.
34. Meschede J, Šadić M, Furthmann N, Miedema T, Sehr DA, Müller-Rischart AK, et al. The parkin-coregulated gene product PACRG promotes TNF signaling by stabilizing LUBAC. Sci Signal. 2020;13:eaav1256.
35. Martínez-Hernández R, Serrano-Somavilla A, Ramos-Leví A, Sampedro-Nunez M, Lens-Pardo A, De Nova JLM, et al. Integrated miRNA and mRNA expression profiling identifies novel targets and pathological mechanisms in autoimmune thyroid diseases. EBioMedicine. 2019;50:329–42.
36. Agirre X, Román‐Gómez J, Vázquez I, Jiménez‐Velasco A, Garate L, Montiel‐Duarte C, et al. Abnormal methylation of the common PARK2 and PACRG promoter is associated with downregulation of gene expression in acute lymphoblastic leukemia and chronic myeloid leukemia. Int J Cancer. 2006;118:1945–53.
37. Li W, Tang W, Teves ME, Zhang Z, Zhang L, Li H. et al. A MEIG1/PACRG complex in the manchette is essential for building the sperm flagella. Development. 2015;142:921–30.
38. Lorenzetti D, Bishop CE, Justice MJ. Deletion of the Parkin coregulated gene causes male sterility in the quakingviable mouse mutant. Proc Natl Acad Sci. 2004;101:8402-07.
39. Dawe HR, Farr H, Portman N, Shaw MK, Gull K. The Parkin co-regulated gene product, PACRG, is an evolutionarily conserved axonemal protein that functions in outer-doublet microtubule morphogenesis. J Cell Sci. 2005;118: 5421–30.
40. Ikeda T. Parkin-co-regulated gene (PACRG) product interacts with tubulin and microtubules. FEBS Lett. 2008;582:1413–8.
41. Wilson GR, Wang HX, Egan GF, Robinson PJ, Delatycki MB, O'Bryan MK, et al. Deletion of the Parkin co-regulated gene causes defects in ependymal ciliary motility and hydrocephalus in the quaking viable mutant mouse. Hum Mol Genet. 2010;19:1593–602.
42. Loucks CM, Bialas NJ, Dekkers MP, Walker DS, Grundy LJ, Li C, et al. PACRG, a protein linked to ciliary motility, mediates cellular signaling. Mol Biol Cell. 2016;27:2133–44.
43. Wilson GR, Sim MLJ, Brody KM, Taylor JM, McLachlan RI, O'Bryan MK, et al. Molecular analysis of the PArkin co-regulated gene and association with male infertility. Fertil Steril. 2010;93:2262–8.
44. Zhang Z, Shen X, Gude DR, Wilkinson BM, Justice MJ, Flickinger CJ, et al. MEIG1 is essential for spermiogenesis in mice. Proc Natl Acad Sci. 2009;106:17055–60.
45. Sweett H, Fonseca PAS, Suárez-Vega A, Livernois A, Miglior F, Cánovas A. Genome-wide association study to identify genomic regions and positional candidate genes associated with male fertility in beef cattle. Sci Rep. 2020;10:1–14.
46. Oud MS, Houston BJ, Volozonoka L, Mastrorosa FK, Holt GS, Alobaidi BKS, et al. Exome sequencing reveals variants in known and novel candidate genes for severe sperm motility disorders. Hum Reprod. 2021;36:2597–611.
47. Kitagawa H, Ray WJ, Glantschnig H, Nantermet PV, Yu Y, Leu CT, et al. A regulatory circuit mediating convergence between Nurr1 transcriptional regulation and Wnt signaling. Mol Cell Biol. 2007;27:7486–96.
48. Bonne A, Vreede L, Kuiper RP, Bodmer D, Jansen C, Eleveld M, et al. Mapping of constitutional translocation breakpoints in renal cell cancer patients: identification of KCNIP4 as a candidate gene. Cancer Genet Cytogenet. 2007;179:11–8.
49. Tang H, Wei P, Duell EJ, Risch HA, Olson SH, Bueno-de-Mesquita HB, et al. Axonal guidance signaling pathway interacting with smoking in modifying the risk of pancreatic cancer: a gene-and pathway-based interaction analysis of GWAS data. Carcinogenesis. 2014;35:1039–45.
50. Horinouchi M, Yagi M, Imanishi H, Mori T, Yanai T, Hayakawa A, et al. Association of genetic polymorphisms with hepatotoxicity in patients with childhood acute lymphoblastic leukemia or lymphoma. Pediatr Hematol Oncol. 2010;27:344–54.
51. Mosley JD, Shaffer CM, Van Driest SL, Weeke PE, Wells QS, Karnes JH, et al. A genome-wide association study identifies variants in KCNIP4 associated with ACE inhibitor-induced cough. Pharmacogenomics J. 2016;16:231–7.
52. Massone S, Vassallo I, Castelnuovo M, Fiorino G, Gatta E, Robello M, et al. RNA polymerase III drives alternative splicing of the potassium channel–interacting protein contributing to brain complexity and neurodegeneration. J Cell Biol. 2011;193:851–66.
53. Weißflog L, Scholz CJ, Jacob CP, Nguyen TT, Zamzow K, Groß-Lesch S, et al. KCNIP4 as a candidate gene for personality disorders and adult ADHD. Eur Neuropsychopharmacol. 2013;23:436–47.
54. Jenkins CA, Kalmar L, Matiasek K, Mari L, Kyöstilä K, Lohi H, et al. Characterisation of canine KCNIP4: A novel gene for cerebellar ataxia identified by whole-genome sequencing two affected Norwegian Buhund dogs. PLoS Genet. 2020;16:e1008527.
55. Ji G, Li S, Ye L, Guan J. Gene Module analysis reveals cell-type specificity and potential target genes in autism’s pathogenesis. Biomedicines. 2021;9:410.
56. Qaiser F, Sadoway T, Yin Y, Zulfiqar Ali Q, Nguyen CM, Shum N, et al. Genome sequencing identifies rare tandem repeat expansions and copy number variants in Lennox–Gastaut syndrome. Brain Commun. 2021;3:fcab207.
57. Himes BE, Sheppard K, Berndt A, Leme AS, Myers RA, Gignoux CR, et al. Integration of mouse and human genome-wide association data identifies KCNIP4 as an asthma gene. PLoS One. 2013;8:e56179.
58. Tran NK, Lea RA, Holland S, Nguyen Q, Raghubar AM, Sutherland HG, et al. Multi-phenotype genome-wide association studies of the Norfolk Island isolate implicate pleiotropic loci involved in chronic kidney disease. Sci Rep. 2021;11:1–10.
59. Su ZJ, Wang XY, Zhou C, Chai Z. Down‐regulation of miR‐3068‐3p enhances kcnip4‐regulated A‐type potassium current to protect against glutamate‐induced excitotoxicity. J Neurochem. 2020;153:617–30.
60. Laugier L, Frade AF, Ferreira FM, Baron MA, Teixeira PC, Cabantous S, et al. Whole-genome cardiac DNA methylation fingerprint and gene expression analysis provide new insights in the pathogenesis of chronic Chagas disease cardiomyopathy. Clin Infect Dis. 2017;65:1103–11.
61. Smith JL, Wilson ML, Nilson SM, Rowan TN, Schnabel RD, Decker JE, Seabury CM. Genome-wide association and genotype by environment interactions for growth traits in US Red Angus cattle. BMC Genomics. 2022;23:1–22.
62. Jin CF, Chen YJ, Yang ZQ, Shi K, Chen CK. A genome-wide association study of growth trait-related single nucleotide polymorphisms in Chinese Yancheng chickens. Genet Mol Res. 2015;14:15783–92.
63. Cha J, Choo H, Srikanth K, Lee SH, Son JW, Park MR, et al. Genome-Wide Association Study Identifies 12 Loci Associated with Body Weight at Age 8 Weeks in Korean Native Chickens. Genes. 2021;12:1170.
64. Wang S, Wang Y, Li Y, Xiao F, Guo H, Gao H, et al. Genome-Wide Association Study and Selective Sweep Analysis Reveal the Genetic Architecture of Body Weights in a Chicken F2 Resource Population. Front Vet Sci. 2022;9: 875454
65. Fan QC, Wu PF, Dai GJ, Zhang GX, Zhang T, Xue Q, et al. Identification of 19 loci for reproductive traits in a local Chinese chicken by genome-wide study. Genet Mol Res. 2017;16:1–8.
