# Supplementary material for: A comprehensive analysis of the genetic diversity and environmental adaptability in worldwide Merino and Merino-derived sheep breeds
Source: Genet Sel Evol. 2023 Apr 3;55:24. doi: 10.1186/s12711-023-00797-z (PMC10069132; doi:10.1186/s12711-023-00797-z)
Supplement: Supplementary file 16 — Additional file 16: Table S7. Description of gene functions highlighted in the GeneMANIA network. [file 12711_2023_797_MOESM16_ESM.docx]

**Additional file 16: Table S7.** Description of gene functions highlighted in the GeneMANIA network.

| **Function** | **FDR** | **Genes in network** | **Genes in genome** |
| --- | --- | --- | --- |
| Pore complex | 1.98E-07 | 6 | 11 |
| Regulation of humoral immune response | 1.33E-05 | 8 | 61 |
| Complement activation | 1.91E-05 | 8 | 67 |
| Integral component of plasma membrane | 0.0030 | 11 | 295 |
| Positive regulation of protein serine/threonine kinase activity | 0.0093 | 9 | 212 |
| Humoral immune response | 0.0398 | 8 | 197 |
| Negative regulation of leukocyte migration | 0.1054 | 4 | 35 |
| Substrate-dependent cell migration | 0.1054 | 3 | 13 |
| Positive regulation of MAP kinase activity | 0.3571 | 6 | 149 |
| Activation of protein kinase activity | 0.4266 | 7 | 223 |
